# Multiple Roles for the Non-Coding RNA SRA in Regulation of Adipogenesis and Insulin Sensitivity

**DOI:** 10.1371/journal.pone.0014199

**Published:** 2010-12-02

**Authors:** Bin Xu, Isabelle Gerin, Hongzhi Miao, Dang Vu-Phan, Craig N. Johnson, Ruichuan Xu, Xiao-Wei Chen, William P. Cawthorn, Ormond A. MacDougald, Ronald J. Koenig

**Affiliations:** 1 Division of Metabolism, Endocrinology and Diabetes, Department of Internal Medicine, University of Michigan Medical Center, Ann Arbor, Michigan, United States of America; 2 Department of Molecular and Integrative Physiology, University of Michigan Medical Center, Ann Arbor, Michigan, United States of America; 3 Department of Pediatrics, University of Michigan Medical Center, Ann Arbor, Michigan, United States of America; 4 Cellular and Molecular Biology Graduate Program, University of Michigan Medical Center, Ann Arbor, Michigan, United States of America; 5 Microarray Core, Comprehensive Cancer Center, University of Michigan Medical Center, Ann Arbor, Michigan, United States of America; 6 Life Sciences Institute, University of Michigan, Ann Arbor, Michigan, United States of America; University of Padova, Italy

## Abstract

Peroxisome proliferator-activated receptor-γ (PPARγ) is a master transcriptional regulator of adipogenesis. Hence, the identification of PPARγ coactivators should help reveal mechanisms controlling gene expression in adipose tissue development and physiology. We show that the non-coding RNA, Steroid receptor RNA Activator (SRA), associates with PPARγ and coactivates PPARγ-dependent reporter gene expression. Overexpression of SRA in ST2 mesenchymal precursor cells promotes their differentiation into adipocytes. Conversely, knockdown of endogenous SRA inhibits 3T3-L1 preadipocyte differentiation. Microarray analysis reveals hundreds of SRA-responsive genes in adipocytes, including genes involved in the cell cycle, and insulin and TNFα signaling pathways. Some functions of SRA may involve mechanisms other than coactivation of PPARγ. SRA in adipocytes increases both glucose uptake and phosphorylation of Akt and FOXO1 in response to insulin. SRA promotes S-phase entry during mitotic clonal expansion, decreases expression of the cyclin-dependent kinase inhibitors p21Cip1 and p27Kip1, and increases phosphorylation of Cdk1/Cdc2. SRA also inhibits the expression of adipocyte-related inflammatory genes and TNFα-induced phosphorylation of c-Jun NH_2_-terminal kinase. In conclusion, SRA enhances adipogenesis and adipocyte function through multiple pathways.

## Introduction

Obesity is a prevalent health hazard closely associated with a number of pathological disorders, including type 2 diabetes, cardiovascular disease, hypertension, cancer, and gallbladder disease. Adipocytes play a central role in energy balance, both as reservoirs of fuel and as endocrine cells, secreting factors (adipokines) that regulate whole body energy metabolism and glucose homeostasis [1]. Adipogenesis is a complex process that is highly regulated by positive and negative stimuli, including a variety of hormones and nutritional signals [2], [3], [4], [5]. Adipocyte differentiation is commonly studied in immortalized cell lines such as 3T3-L1 preadipocytes [2], [4], [6] and the pluripotent bone marrow-derived mesenchymal cell line ST2 [7], both of which can be differentiated into mature adipocytes by standard hormone cocktails. During adipogenesis, fibroblast-like preadipocytes differentiate into lipid-laden and insulin-responsive adipocytes. This process occurs in several stages (growth arrest, mitotic clonal expansion and terminal differentiation) and is driven by the coordinated effects of a number of transcription factors and signaling molecules, including peroxisome proliferator-activated receptor gamma (PPARγ), the CCAAT/enhancer-binding proteins (C/EBPs) [4], [8], Kruppel-like factors (KLFs) [9], [10], Wingless proteins (Wnt) [11], [12], GATA2 [13], [14] and cell cycle proteins [15], [16], [17].

Transcription factors function in part by recruiting coregulators that epigenetically remodel chromatin and/or bridge the complexes in which they reside to the basal transcriptional machinery. Some coregulators important in adipogenesis have essential enzymatic activities, such as the SW1/SNF complex that controls ATP-dependent chromatin remodeling [18], [19], and the histone acetyltransferase proteins CBP and p300 [20], [21]. Others, such as the p160 family of coactivators, SRC-1, TIF2/SRC-2 and AIB1/SRC-3, function as scaffolds, although they also have some histone acetyltransferase activity [22], [23], [24]. Conversely, corepressors such as nuclear receptor corepressor (NCoR) and silencing mediator of retinoid and thyroid hormone receptors (SMRT) recruit histone deacetylases to target promoters, and therefore are anti-adipogenic [25].

The steroid receptor RNA activator (SRA) is a unique coregulator that functions as a non-coding RNA [26], although incorporation of an additional 5′ region can result in translation of an SRA protein (SRAP) that also has coactivator activity [27], [28]. SRA was initially shown to enhance gene expression through a ribonucleoprotein complex with steroid receptors and SRC-1 [26]. SRA also functions as an RNA coactivator for thyroid hormone receptors (TRs) [29], [30], retinoic acid receptors (RARs) [30] and the muscle cell differentiation factor MyoD [31]. In addition, SRA may act as an RNA scaffold for corepressor complexes [32], [33]. A potential role for SRA in adipogenesis has yet to be explored.

In this study, we report that SRA binds to PPARγ *in vitro* and in 3T3-L1 adipocytes, and enhances PPARγ transcriptional activity. SRA promotes adipocyte differentiation; up-regulates the expression of PPARγ, C/EBPα and other adipocyte genes; and increases glucose uptake and phosphorylation of Akt and FOXO1 in response to insulin. To uncover mechanism(s) by which SRA regulates adipocyte function, we identified SRA responsive genes by gene expression profiling analysis of SRA overexpressing ST2 cells and SRA knockdown 3T3-L1 cells that had been differentiated into mature adipocytes. The data show that SRA regulates gene expression networks in various cellular processes, including the cell cycle and insulin related signal transduction pathways. Indeed, SRA promotes S-phase entry during mitotic clonal expansion and inhibits phosphorylation of c-Jun NH_2_-terminal kinase (JNK) in response to tumor necrosis factor-α (TNFα) signaling.

## Results

### The non-coding steroid receptor RNA activator (SRA) is a transcriptional coactivator of PPARγ

SRA was initially identified as a coactivator of steroid receptors [26] and has subsequently been shown to coactivate several nonsteroid nuclear hormone receptors such as thyroid hormone receptors [29], RARs [30] and SF-1 [34]. SRA is enriched in liver, skeletal muscle [26] and adipose tissue (described below), which are important organs for controlling whole body energy balance, glucose homeostasis and insulin sensitivity. Therefore, we tested whether SRA binds to and coactivates PPARγ, a critical transcriptional regulator of these processes.

**Figure 1:**
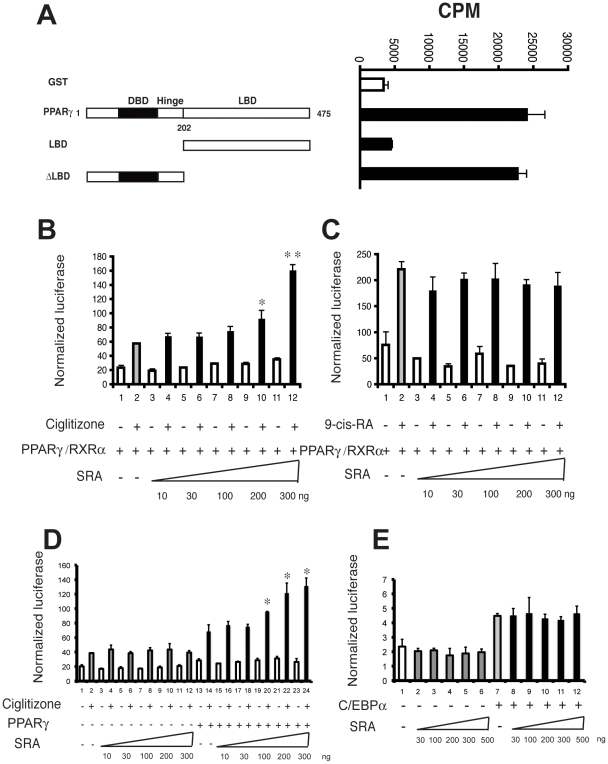
SRA binds to PPARγ in vitro and enhances its transcriptional activity. A, Schematic presentation of full length PPARγ and its truncation mutants used for *in vitro* RNA binding studies. Purified full-length GST, GST-PPARγ, and truncation proteins GST-PPARγLBD and GST-PPARγΔLBD bound to glutathione agarose beads were incubated with a ^32^P-labeled SRA (RNA) probe. After washing, the radioactivity associated with the beads was determined by liquid scintillation counting. B, JEG cells were transfected with 1 ng of PPARγ and RXRα 10 ng of the internal control vector pRL-TK-*Renilla*, 200 ng PPRE-luc, and increasing doses of SRA as indicated. One day post-transfection, the cells were treated with or without ciglitizone, and cells were harvested for firefly and *Renilla* luciferase assays after an additional day. C. Similar transfection to B, but cells were treated with the RXR ligand 9-cis-RA. D, Similar transfection to B, but without cotransfection of RXR. E, JEG cells were transfected with 50 ng of p42C/EBPα 1 ng of the internal control vector pRL-CMV570-*Renilla*, 150 ng pObluc-760-luc, and increasing doses of SRA as indicated. 48 hr post-transfection, cells were harvested for firefly and *Renilla* luciferase assays. For B, C, D and E, the *y* axis represents arbitrary firefly luciferase units normalized to *Renilla* luciferase values. Experiments were performed with triplicate samples and were repeated three times. Error bars indicate standard deviations. The results of statistical analyses by analysis of variance (ANOVA) followed by Scheffe's test, comparing bar 2 without SRA transfection under ciglitizone induction with bars with SRA transfections and ciglitizone inductions (* p<0.05 and ** p<0.01).

As shown in Figure 1A, SRA binds to full length PPARγ *in vitro*. To identify the protein region responsible for RNA binding, we tested PPARγ deletion mutants. This revealed that the PPARγ ligand binding domain (LBD) does not bind to SRA, and the N-terminal half of PPARγ accounts for the full SRA-binding capacity (ΔLBD). This result is consistent with the SRA binding properties of thyroid hormone receptors and SF-1 [29], [34], [35]. The finding that SRA binds to PPARγ *in vitro* prompted us to test for its functional relevance to PPARγ target gene transcription. To this end, a reporter plasmid containing three copies of the acyl-CoA oxidase PPAR response element linked to the luciferase gene (PPRE-luc) was co-transfected with PPARγ, RXRα, pRL-TK *Renilla* luciferase as an internal control, and increasing quantities of SRA in JEG-3 cells. PPARγ ligand (ciglitizone)-dependent transactivation of PPRE-luc was increased by SRA in a dose-dependent manner (Figure 1B). However, RXRα ligand (9-cis-RA)-dependent transactivation of the same reporter construct was not affected by SRA (Figure 1C). SRA also coactivated PPARγ/ciglitazone-dependent luciferase expression in the absence of cotransfected RXR (Figure 1D). In contrast, SRA did not coactivate C/EBPα induction of a leptin promoter-luciferase vector (Figure 1E).

### SRA expression is induced during adipocyte differentiation and SRA is highly expressed in white adipose tissue

**Figure 2:**
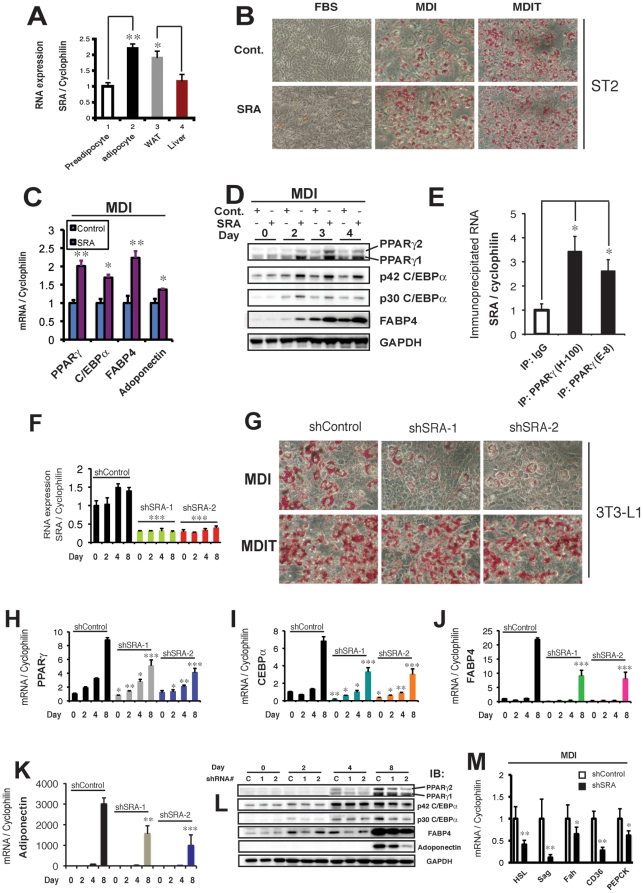
SRA is highly expressed in adipocytes and is pro-adipogenic. A, RT-qPCR was performed to measure SRA RNA levels in 3T3-L1 preadipocytes, mature 3T3-L1 adipocytes, mouse white adipose tissue (WAT) and mouse liver. RNA expression was normalized to cyclophilin mRNA, and expression is given relative to SRA in 3T3-L1 preadipocytes set at 1. B, Control (pMSCV empty vector; *Cont.*) or SRA overexpressing ST2 cells were grown to confluence and treated with FBS, MDI or MDIT to induce adipocyte differentiation. On day 4 of differentiation, cells were stained with Oil Red O to identify triglyceride droplets. C and D, Control and SRA overexpressing ST2 cells were induced by MDI to differentiate into adipocytes. The mRNA (C) or protein levels (D) of adipocyte marker genes were determined by RT-qPCR or immunoblotting, respectively. E, 3T3-L1 cells were differentiated into adipocytes with MDIT. Cell lysates from day 8 of differentiation were immunoprecipitated using two PPARγ antibodies as indicated or normal IgG as a negative control. The immunoprecipitates were subjected to RT-qPCR with primers for mouse SRA or cyclophilin RNA as a non-specific control. The ratio of SRA/cyclophilin in the immunoprecipitates with normal IgG was set at 1. F-L, 3T3-L1 preadipocytes were infected with retroviruses expressing either a control short hairpin RNA (shControl) or one of two shRNAs directed against mouse SRA target sequences (shSRA-1 or -2). Preadipocytes were induced to differentiate with MDI, except MDIT was used for G, lower panel. F, Time course of SRA RNA expression normalized to cyclophilin. G, At day 8 of differentiation, cells were stained with Oil Red O to assess lipid accumulation. H–K, Time course of mRNA expression for adipocyte marker genes PPARγ (H), C/EBPα (I), FABP4/aP2 (J), and adiponectin (K) by RT-qPCR. Data were normalized to cyclophilin and expression of each gene at day 0 was set to 1. L, Time course of adipocyte marker protein expression by immunoblotting. M, shControl or shSRA-2 3T3-L1 preadipocytes were differentiated with MDI. At day 8 of differentiation, RT-qPCR was used to measure relative mRNA expression levels for PPARγ target genes, normalized to cyclophilin. The average normalized expression of each target mRNA in control cells is set at 1. Data are given as the mean ± S.D. Statistical significance was evaluated with Student's t test: * p<0.05, ** p<0.01 and *** p<0.001. These results are representative of three independent experiments.

SRA expression was induced ∼2 fold when 3T3-L1 preadipocytes differentiated into mature adipocytes (Figure 2A). In addition, we found SRA to be more highly expressed in mouse white adipose tissue (WAT) than liver, an organ previously shown to express SRA at high levels [26]. These data suggest that SRA may play an important role in adipocyte biology.

### SRA overexpression promotes differentiation of adipocyte precursor ST2 cells

To probe whether SRA can modulate adipogenesis, we used marrow-derived mesenchymal ST2 cells as an *in vitro* model. SRA RNA was overexpressed ∼140 fold through retroviral infection with pMSCV-SRA (data not shown). Confluent, growth-arrested ST2 adipocyte precursors were treated with fetal bovine serum (FBS) or were induced to differentiate with a hormone cocktail containing 3-isobutyl-1-methylxanthine, dexamethasone, and insulin, denoted MDI, or troglitazone was added to the hormone cocktail (MDIT). At day 4 of differentiation, Oil Red O staining was used to visualize lipid droplets, which characterize mature adipocytes. As shown in Figures 2B and S1A, compared to control ST2 cells that were infected with pMSCV empty vector, SRA overexpression promoted adipogenesis slightly when no differentiation cocktail was used (FBS), and more markedly with the differentiation cocktail MDI. MDIT is a more powerful differentiation cocktail, and hence little additional triacylglycerol accumulation was seen with SRA overexpression in MDIT-treated ST2 cells (Figures 2B and S1A). We used RT-qPCR and immunoblotting to confirm the positive effect of SRA on expression of adipocyte marker genes in ST2 cells differentiated with MDI. As shown in Figure 2C, after 4 days of differentiation, SRA overexpression significantly enhanced the mRNA expression of adipocyte master regulators PPARγ and C/EBPα, as well as the PPARγ target gene fatty acid binding protein 4 (FABP4/aP2) and the adipocyte specific gene adiponectin. Consistent with these effects on mRNA expression, SRA also increased the protein expression of adipocyte markers (Figure 2D). Time course data indicate that from day 2 of differentiation, SRA increased protein expression ∼2 fold over control cells for PPARγ1, PPARγ2, and p42 and p30 C/EBPα, and more than 3 fold for FABP4/aP2 (Figure 2D). These results suggest that SRA is a pro-adipogenic factor.

### SRA knockdown inhibits the differentiation of 3T3-L1 preadipocytes

Since the endogenous SRA level in 3T3-L1 cells is 2-3 fold higher than in ST2 cells (data not shown), we used 3T3-L1 cells to evaluate the effects of SRA depletion on adipogenesis. First, we tested whether endogenous SRA is associated with PPARγ in adipocytes. RNA-protein co-immunoprecipitation was performed from fully differentiated 3T3-L1 adipocytes using either of two different PPARγ antibodies or normal IgG as a negative control. RNA was isolated from the immunoprecipitates and RT-qPCR was performed to quantify the immunoprecipitated SRA. The results indicate that endogenous SRA RNA coimmunoprecipitates with PPARγ (Figure 2E), demonstrating a physical interaction between these molecules within adipocytes.

Next, endogenous SRA was knocked down in subconfluent 3T3-L1 preadipocytes with retroviral expression of short hairpin RNAs (shRNA) against SRA. Two shRNAs (shSRA-1, -2) with different targeting sequences in mouse SRA1 were designed and transduced into 3T3-L1 preadipocytres. Preadipocytes stably infected with retroviral shSRAs or shControl were then induced to differentiate with MDI, and samples from different time points were collected for RNA or protein analysis. Both shSRAs efficiently knocked down endogenous SRA to ∼20% of control at day 0 and throughout differentiation (Figure 2F). We then investigated the effects of SRA knockdown on adipocyte differentiation. As shown in Figures 2G and S1B, on day 8 of differentiation with MDI induction, lipid accumulation (Oil Red O staining) was dramatically decreased in adipocytes harboring shSRA-1 or -2. In contrast, little if any decreased triacylglycerol accumulation was seen with SRA knockdown in MDIT-treated 3T3-L1 cells (Figures 2G and S1B), presumably because this strong differentiation cocktail largely relieves the dependence on SRA for adipocyte differentiation.

The inhibitory effect of SRA knockdown on MDI-induced adipogenesis was studied further at the RNA and protein levels. PPARγ and C/EBPα mRNAs were significantly decreased in day 8 adipocytes with endogenous SRA knockdown by shSRA-1 or -2, compared to shControl (Figures 2H and 2I). Small but significant decreases in PPARγ mRNA were found as early as day 2 of adipogenesis, and C/EBPα mRNA levels were inhibited even on day 0. As expected from decreased adipogenic transcription factor expression, upregulation of the adipocyte markers FABP4/aP2 and adiponectin was significantly inhibited by both shSRA-1 and -2 (Figure 2J and 2K). Immunoblotting analysis confirmed these effects of SRA knockdown at the protein level (Figure 2L). In SRA knockdown cells, inhibition of PPARγ protein expression was observed beginning at day 4 of differentiation compared to shControl cells. The inhibitory effect of SRA knockdown on C/EBPα protein expression was most apparent at days 2 and 8. FABP4/aP2 protein expression was inhibited from day 2 onward. Furthermore, protein expression of adiponectin, an anti-inflammatory adipokine that was detected only on day 8, also was inhibited by SRA knockdown. In addition to FABP4/aP2 (Figure 2J), the mRNA expression of various other PPARγ target genes also was decreased by SRA knockdown (Figure 2M).

### Genome-wide identification of SRA responsive genes

**Figure 3:**
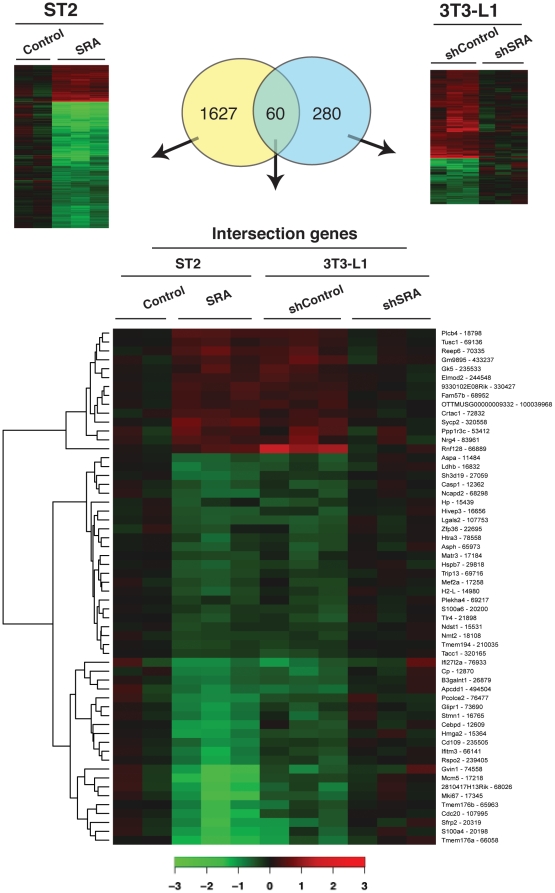
Microarray analysis of mRNA expression of SRA responsive genes. Total RNA was isolated from fully differentiated (MDIT day 4) SRA overexpressing and control ST2 adipocytes, or fully differentiated (MDIT day 8) shSRA knockdown or shControl 3T3-L1 adipocytes. Gene expression analysis was performed using Affymetrix mouse genome 430 2.0 arrays. The Venn diagram shows the number of genes with significant expression changes in SRA overexpressing versus control ST2 adipocytes (left, yellow), genes that changed in endogenous SRA knockdown versus control 3T3-L1 adipocytes (right, blue), and the intersection genes. Heat maps are shown for Log_2_ expression changes of SRA responsive genes corresponding to the above comparisons. Increased or decreased mRNA expression is represented by red or green color, respectively.

To gain further insight into the functions of SRA in adipocyte biology, which may extend beyond coactivation of PPARγ, we performed an Affymetrix GeneChip microarray analysis to identify SRA responsive genes in SRA overexpressing ST2 adipocytes and SRA knockdown 3T3-L1 adipocytes, versus their respective control cells. These experiments utilized cells that had been maximally differentiated with MDIT (4 days for ST2 and 8 for 3T3-L1), since under those conditions SRA overexpression or knockdown has relatively little effect on adipogenesis (Figures 2B, 2G and S1). Thus, this experimental design is more likely to reveal effects of SRA in mature adipocytes beyond its ability to enhance adipogenesis via PPARγ coactivation. As shown in Figure 3, we first identified genes whose expression was significantly changed in ST2 adipocytes overexpressing SRA compared to controls. A total of 1687 genes/expressed-sequence tags (ESTs) were significantly altered (p<0.05). Among these, 421 were up-regulated and 1266 were down-regulated by SRA overexpression. For 3T3-L1 adipocytes, the expression of 340 genes/ESTs was altered by endogenous SRA knockdown (145 up, 195 down). Sixty genes/ESTs (intersection genes) were commonly regulated (and in opposite directions) by SRA overexpression in ST2 adipocytes and endogenous SRA knockdown in 3T3-L1 adipocytes. Heat maps representing gene expression levels are shown in Figure 3, and the complete data set has been deposited in the Gene Expression Omnibus (accession number GSE21594).

The 1687 genes regulated by SRA overexpression, the 340 genes regulated by SRA knockdown, and the 60 intersection genes were each analyzed for over-representation of Gene Ontology (GO) biological processes (BP) and molecular functions (MF). The most overrepresented GO terms are listed in Tables 1, 2, 3, 4, 5, 6. Full listings of overrepresented GO terms are provided in Tables S1, S2, S3, S4, and listings of the significantly changed genes associated with these GO terms are provided in Data Sets S1, S2, S3. Selected GO term findings are presented and expanded upon below.

**Table 1:** Top six GO terms in biological processes (BP) overrepresented amongst genes with altered expression in SRA overexpressing versus empty vector control ST2 adipocytes.

GO BP ID	Pvalue	ExpCount	Count	Size	Term
GO:0000278	<0.001	26	58	153	mitotic cell cycle
GO:0007067	<0.001	20	46	115	mitosis
GO:0006955	<0.001	32	64	188	immune response
GO:0007155	<0.001	47	85	278	cell adhesion
GO:0051301	<0.001	29	57	169	cell division
GO:0000279	<0.001	25	51	146	M phase

**Table 2:** Top six GO terms in molecular function (MF) overrepresented amongst genes with altered expression in SRA overexpressing versus empty vector control ST2 adipocytes.

GO MF ID	Pvalue	ExpCount	Count	Size	Term
GO:0005509	<0.001	65	102	393	calcium ion binding
GO:0004872	<0.001	56	85	339	receptor activity
GO:0003779	<0.001	23	41	138	actin binding
GO:0060089	<0.001	115	151	691	molecular transducer activity
GO:0030020	<0.001	3	10	18	extracellular matrix structural constituent conferring tensile strength
GO:0003924	0.001	16	29	95	GTPase activity

**Table 3:** Top six GO terms in biological processes (BP) overrepresented amongst genes with altered expression in endogenous SRA knockdown versus control 3T3-L1 adipocytes.

GO BP ID	Pvalue	ExpCount	Count	Size	Term
GO:0007155	<0.001	8	21	278	cell adhesion
GO:0006817	<0.001	1	6	28	phosphate transport
GO:0030199	<0.001	0	4	12	collagen fibril organization
GO:0007169	<0.001	3	10	92	transmembrane receptor protein tyrosine kinase signaling pathway
GO:0048675	<0.001	0	4	13	axon extension
GO:0007275	<0.001	28	45	978	multicellular organismal development

**Table 4:** Top six GO terms in molecular function (MF) overrepresented amongst genes with altered expression in endogenous SRA knockdown versus control 3T3-L1 adipocytes.

GO MF ID	Pvalue	ExpCount	Count	Size	Term
GO:0004872	<0.001	17	34	588	receptor activity
GO:0048503	<0.001	1	8	51	GPI anchor binding
GO:0019199	<0.001	1	6	36	transmembrane receptor protein kinase activity
GO:0060089	0.001	20	35	691	molecular transducer activity
GO:0005543	0.001	3	10	101	phospholipid binding
GO:0030020	0.001	1	4	18	extracellular matrix structural constituent conferring tensile strength

**Table 5:** GO terms in biological processes (BP) overrepresented amongst the intersection genes.

GO BP ID	Pvalue	ExpCount	Count	Size	Term
GO:0006954	0.002	1	4	90	inflammatory response
GO:0000271	0.002	0	2	13	polysaccharide biosynthetic process
GO:0051301	0.002	1	5	169	cell division
GO:0042035	0.008	0	2	24	regulation of cytokine biosynthetic process
GO:0000279	0.009	1	4	146	M phase
GO:0019882	0.01	0	2	27	antigen processing and presentation
GO:0009607	0.01	0	3	80	response to biotic stimulus

**Table 6:** GO terms in molecular function (MF) overrepresented amongst the intersection genes.

GO MF ID	Pvalue	ExpCount	Count	Size	Term
GO:0005520	0.003	0	2	16	insulin-like growth factor binding

### SRA promotes insulin sensitivity and insulin-stimulated glucose uptake in adipocytes, and increases phosphorylation of Akt and FOXO1 in response to insulin

**Figure 4:**
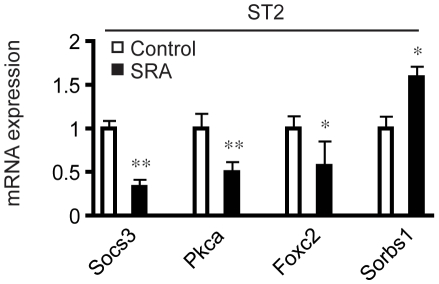
RT-qPCR analyses confirm expression changes of genes related to insulin sensitivity in SRA overexpressing ST2 adipocytes. Total RNA was isolated from day 4 MDIT-differentiated SRA overexpressing or empty vector control ST2 cells. Gene expression was then analyzed by RT-qPCR and was normalized to the expression of cyclophilin. Significance was determined by Student's t-test compared to each control. * P<0.05, **P<0.01. Experiments were repeated at least three times with triplicate samples.

**Figure 5:**
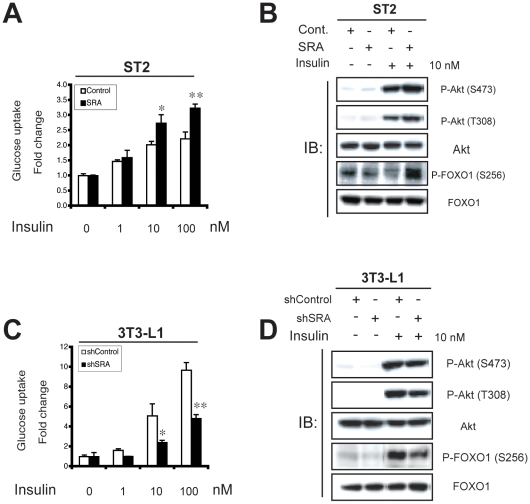
SRA increases insulin-stimulated glucose uptake and phosphorylation of Akt and FOXO1 in mature adipocytes. A, ST2 preadipocytes infected with empty vector pMSCV (control) or pMSCV-SRA were differentiated with MDIT. On day 5 of differentiation, adipocytes were serum-starved for 3 h and insulin-stimulated glucose uptake was quantified using 2-deoxy-D-[^14^C]glucose. Data were normalized to total protein, and results are presented as fold change compared to basal levels without insulin treatment. Data are given as the mean ± S.D. Statistical significance was evaluated with Student's *t*-test: * p<0.05 and ** p<0.01. B, ST2 control (pMSCV) and SRA overexpressing (pMSCV-SRA) ST2 preadipocytes were differentiated as in A. At the end of day 4 of differentiation, adipocytes were serum-starved for 12 h, cells were then treated without or with 10 nM insulin for 10 min. Total cell lysates were subjected to immunoblotting (IB) with anti-phospho-Akt and phospho-FOXO1 antibodies as indicated. The membrances were then stripped and reprobed with antibodies for total Akt and FOXO1. C, A similar experiment of insulin-stimulated glucose uptake was performed on day 11 of differentiation by MDIT in 3T3-L1 cells infected with retroviruses expressing either a control shRNA (shControl) or an shRNA against SRA (shSRA). D, Control (shControl) and SRA knockdown (shSRA) 3T3-L1 preadipocytes were similarly differentiated as in C. Cells were serum-starved for 12 h, and then treated without or with 10 nM insulin for 20 min. Similar immunoblottings to B were performed with antibodies as indicated. These results are representative of at least three independent experiments.

The GO term Insulin Receptor Signaling Pathway was overrepresented in the gene list from SRA overexpressing ST2 cells (Table S1). Elevated SRA repressed the expression of several genes within this GO term that function as negative regulators of insulin sensitivity (Table 7), including suppressor of cytokine signaling (SOCS) -1 and -3, protein kinase C-alpha (Prkca) and forkhead box C2 (Foxc2) [36], [37], [38], [39]. In contrast, SRA overexpression increased the expression of sorbin and SH3 domain containing 1 (Sorbs1), which encodes Cbl-associated protein (CAP), part of a phosphatidylinositol 3-kinase-independent pathway for insulin-stimulated translocation of the glucose transporter GLUT4 [40]. These results were confirmed by RT-qPCR (Figure 4). Given these data, the ability of SRA to promote insulin-stimulated glucose uptake was examined in differentiated adipocytes. We wished to assess whether the postulated ability of SRA to enhance this insulin action is simply a marker of better differentiation, or whether SRA might have a specific effect on insulin-stimulated glucose uptake beyond its general ability to enhance differentiation. Therefore, we differentiated preadipocytes with MDIT rather than MDI, since, as noted previously, under MDIT conditions the expression level of SRA has relatively little effect on adipocyte differentiation (Figures 2B, 2G and S1). Indeed, our data demonstrate that fully differentiated ST2 adipocytes overexpressing SRA have increased insulin-stimulated glucose uptake (Figure 5A). Consistent with these data, SRA overexpression also was associated with increased insulin-stimulated phosphorylation of Akt at both Ser-473 and Thr-308, as well as increased phosphorylation of FOXO1, a downstream target of Akt signaling (Figure 5B). Conversely, fully differentiated 3T3-L1 adipocytes with knockdown of endogenous SRA had decreased insulin-stimulated glucose uptake (Figure 5C) and decreased phosphorylation of Akt and FOXO1 (Figure 5D). These data support the hypothesis that SRA not only regulates adipogenesis, but also enhances insulin signaling and glucose uptake.

**Table 7:** Genes associated with the GO term insulin receptor signaling pathway that have significantly altered expression in SRA overexpressing ST2 cells.

Probe set	Symbol	Gene name	p-value	Fold change*
**Insulin receptor signaling pathway**				
1455899_x_at	Socs3	suppressor of cytokine signaling 3	<0.001	−1.64
1450945_at	Prkca	protein kinase C, alpha	<0.001	−0.91
1455706_at	Stxbp4	syntaxin binding protein 4	<0.001	−0.82
1416693_at	Foxc2	forkhead box C2	<0.001	−0.55
1450446_a_at	Socs1	suppressor of cytokine signaling 1	0.001	−0.43
1425098_at	Zfp106	zinc finger protein 106	0.002	0.5
1444472_at	Snf1lk2	SNF1-like kinase 2	0.001	0.6
1443983_at	Sorbs1	sorbin and SH3 domain containing 1	<0.001	0.74

*Fold change log_2_ format.

### Cell cycle genes are reciprocally regulated by SRA overexpression in ST2 adipocytes and SRA knockdown in 3T3-L1 adipocytes

**Figure 6:**
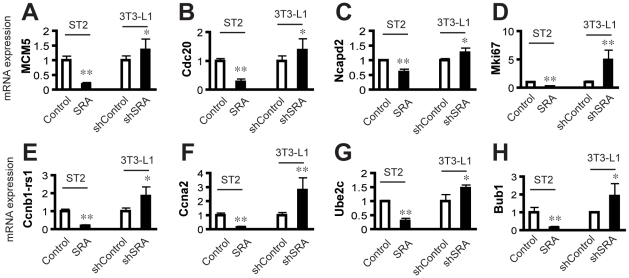
RT-qPCR analyses confirm expression changes of SRA responsive cell cycle related genes. Total RNA was isolated from day 4 MDIT-differentiated SRA overexpressing or empty vector control ST2 cells. Similarly, total RNA was isolated from day 8 MDIT-differentiated 3T3-L1 SRA knockdown (shSRA) or control (shControl) adipocytes. Gene expression was then analyzed by RT-qPCR, normalized to cyclophilin. Significance of data was determined by Student's *t*-test compared to each control. * P<0.05, **P<0.01. Experiments were repeated at least three times with triplicate samples.

Two of the top 5 intersection gene GO-BP terms are Cell Division and M Phase (Table 5). Within these GO terms, the cell cycle and DNA replication-associated genes Mcm5, Cdc20, Tacc1, Mki67 and Ncapd2 were significantly down-regulated by SRA overexpression in ST2 cells and up-regulated by endogenous SRA knockdown in 3T3-L1 cells (Figure 3 and Data Set S3). RT-qPCR was used to confirm these changes for Mcm5, Cdc20, Ncapd2 and Mki67 (Figure 6A–6D). Similarly, 4 of the 6 most enriched GO-BP terms in SRA overexpressing cells were related to the cell cycle, cell division and mitosis (Table 1). The genes associated with the GO terms Mitotic Cell Cycle and DNA Replication Initiation that are most significantly regulated by SRA overexpression are listed in Table 8. Among these, several cyclin genes (Ccnb2, Ccnb1-rs1 and Ccna2), cell cycle-related genes (Ereg, Birc5, Ube2c, Bub1, Cep55) and cell division genes (Cdca5 and Cdc20) were down-regulated more than 2 fold by SRA overexpression. The repression of Ccnb1-rs1, Ccna2, Ube2c and Bub1 by SRA overexpression was confirmed by RT-qPCR, which also demonstrated that these genes are induced by SRA knockdown in 3T3-L1 cells (Figure 6E–6H). Overall, these data suggest that SRA plays a modulatory role in promoting withdrawal from the cell cycle during the final stages of adipogenesis.

**Table 8:** Genes associated with the GO terms Mitotic cell cycle and DNA replication initiation that have significantly altered expression in SRA overexpressing ST2 cells.

Probe set	Symbol	Gene name	p-value	Fold change*
**Mitotic Cell cycle**				
1419431_at	Ereg	epiregulin	<0.001	−2.34
1424278_a_at	Birc5	baculoviral IAP repeat-containing 5	<0.001	−2.26
1452954_at	Ube2c	ubiquitin-conjugating enzyme E2C	<0.001	−2.23
1450920_at	Ccnb2	cyclin B2	<0.001	−2.21
1424046_at	Bub1	budding uninhibited by benzimidazoles 1 homolog (S. cerevisiae)	<0.001	−2.08
1452242_at	Cep55	centrosomal protein 55	<0.001	−1.99
1416076_at	Ccnb1-rs1	cyclin B1, related sequence 1	<0.001	−1.97
1417911_at	Ccna2	cyclin A2	<0.001	−1.93
1416309_at	Nusap1	nucleolar and spindle associated protein 1	<0.001	−1.84
1416802_a_at	Cdca5	cell division cycle associated 5	<0.001	−1.77
1416664_at	Cdc20	cell division cycle 20 homolog (S. cerevisiae)	<0.001	−1.32
1417457_at	Cks2	CDC28 protein kinase regulatory subunit 2	<0.001	−1.31
1455081_at	Txnl4b	thioredoxin-like 4B	0.001	0.43
1452798_s_at	Mapk1ip1	mitogen activated protein kinase 1 interacting protein 1	0.001	0.49
1422477_at	Cables1	Cdk5 and Abl enzyme substrate 1	<0.001	0.55
1417850_at	Rb1	retinoblastoma 1	<0.001	0.6
**DNA replication initiation**				
1415945_at	Mcm5	minichromosome maintenance deficient 5, cell division cycle 46 (S. cerevisiae)	<0.001	−1.94
1438852_x_at	Mcm6	minichromosome maintenance deficient 6 (MIS5 homolog, S. pombe) (S. cerevisiae)	<0.001	−1.8
1420028_s_at	Mcm3	minichromosome maintenance deficient 3 (S. cerevisiae)	<0.001	−1
1436708_x_at	Mcm4	minichromosome maintenance deficient 4 homolog (S. cerevisiae)	0.003	−0.81
1424143_a_at	Cdt1	chromatin licensing and DNA replication factor 1	0.001	−0.75
1456280_at	Clspn	claspin homolog (Xenopus laevis)	<0.001	−0.74

*Fold change log_2_ format.

### SRA regulates S-phase entry during mitotic clonal expansion

**Figure 7:**
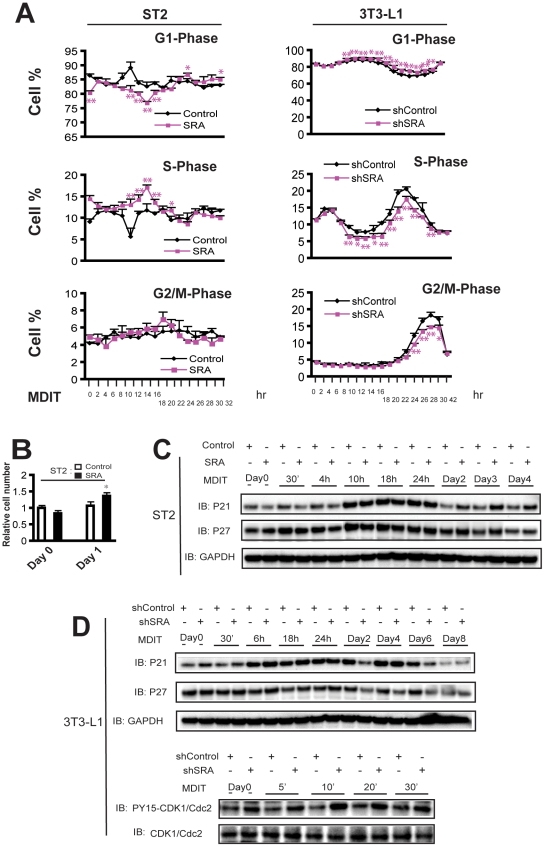
SRA promotes preadipocyte entry into S-phase during mitotic clonal expansion and regulates cell cycle-related proteins. A, Day 0 growth arrested SRA overexpressing or control ST2 preadipocytes (*left*), and SRA knockdown (shSRA) or shControl 3T3-L1 preadipocytes (*right*), were induced by MDIT. Every two hours post-induction, cells were fixed and stained with propidium iodide, and the percentage of cells in each phase of the cell cycle (G1, S, G2/M) was determined by FACS. The results are expressed as means and SDs for triplicate assays. Significance of SRA overexpression or knockdown compared to controls was determined by Student's t-test. P<0.05, **P<0.01. B, On day 0 before and day 1 (24 h) after induction, cell number was determined by Coulter counter, and the cell numbers relative to day 0 control set at 1 were plotted. Significance was determined by Student's t-test. * P<0.05. C, SRA overexpression down-regulates CDK inhibitors p21Cip1 and p27Kip1 in ST2 preadipocytes during MCE, but up-regulates their expression late in differentiation. Cell lysates were prepared from ST2 preadipocytes or adipocytes with SRA overexpression or control cells as indicated during the time course of differentiation with MDIT. Immunoblotting was performed for p21Cip1, p27Kip1, and GAPDH as a loading control. D, Similar to C except 3T3-L1 preadipocytes with endogenous SRA knockdown (shSRA) or control (shControl) were studied. The phosphorylation of CDK1/Cdc2 at Tyr-15 also was studied by immunoblotting during early MCE. These results are representative of at least three independent experiments.

Reentry into the cell cycle is one of the key events in early adipogenesis. Post-confluent preadipocytes become growth arrested at the G0-to-G1 cell cycle transition due to contact inhibition [41]. Upon hormonal induction, growth-arrested 3T3-L1 preadipocytes synchronously re-enter the cell cycle and undergo several rounds of cell division, known as mitotic clonal expansion (MCE). MCE is an essential step in adipocyte maturation [42] in some preadipocyte models. Given that our microarray analysis revealed SRA-dependent changes in the expression of cell cycle genes in mature adipocytes, we investigated whether SRA regulates cell cycle progression during MCE. Growth arrested SRA overexpressing and control ST2 preadipocytes were induced to differentiate with MDIT. FACS analysis of propidium iodide-stained cells was performed every 2 h to elucidate the cell cycle profile during MCE. Similar studies were performed in endogenous SRA knockdown (shSRA) and shControl 3T3-L1 preadipocytes. As shown in Figure 7A (*left*), SRA overexpression in ST2 preadipocytes promotes re-entry to the cell cycle during MCE. SRA ST2 cells had an S-phase peak 14 h after hormone induction (with a concomitant decrease in G1 cells), followed 4 h later by a G2/M phase peak. These changes were not observed in control ST2 cells. Conversely, stable knockdown of endogenous SRA in 3T3-L1 preadipocytes inhibited S-phase entry and the transition to G2/M phase during MCE post-MDIT (Figure 7A, *right*). SRA overexpression also increased BrdU incorporation in the 10–14 h post-MDIT induction by about 2-fold over control ST2 cells (Figure S2), further confirming the ability of SRA to promote S-phase entry in ST2 preadipocytes. In addition, SRA overexpression increased the cell number by ∼30% 24 h post-MDIT (Figure 7B).

### Cell cycle-related genes are regulated by SRA during MCE and adipocyte maturation

Cell cycle progression from G1 to S phase is dependent on the expression of cyclins and cyclin-dependent kinase (CDKs). In addition, cyclin-dependent kinase inhibitors (CKIs) such as the CIP/KIP protein family can bind to cyclin/CDK complexes to inhibit the G1-S phase transition [43]. Cyclin-dependent kinase inhibitors such as p21Cip1 and p27Kip1 play important roles in adipocyte differentiation during both the progression of MCE and in the final exit from the cell cycle during terminal differentiation [44], [45]. In addition, loss of CKIs *in vivo* produces adipocyte hyperplasia and obesity [46]. Given that our data demonstrate a role for SRA to promote entry into S-phase during MCE, we tested whether SRA regulates the expression of CKIs during preadipocyte differentiation. We found that SRA overexpression inhibits p21Cip1 expression from day 0 (before MDIT induction) through the early time points of MCE (up to 10 h post-MDIT), but subsequently up-regulates p21Cip1 after MCE (24 h) until terminal differentiation on day 4 (Figure 7C). The effect of SRA overexpression on p27Kip1 was qualitatively similar but the magnitude of change was far less (Figure 7C). Conversely, knockdown of endogenous SRA in 3T3-L1 preadipocytes up-regulated p21Cip1 and p27Kip1 protein expression during MCE (30 min, 6 h, 18 h), but down-regulated their expression after MCE (day 2) until terminal differentiation on day 6 (Figure 7D, upper panel). Progress of the mammalian cell cycle is also regulated by phosphorylation of other key proteins. Cdk1/Cdc2 is the catalytic subunit for maturation promoting factor (MPF), which includes the regulatory subunit of cyclin B. After binding to cyclin B, Cdc2 is phosphorylated on Tyr-15, yielding an inactive form of the pre-MPF complex during G2 phase and inhibiting cell entry into M phase [47], [48]. Knockdown of endogenous SRA in 3T3-L1 preadipocytes up-regulated the phosphorylation of Tyr-15 on Cdc2 during MCE (Figure 7D; lower panel). Thus, the effects of SRA on the expression of p21Cip1 and p27Kip1, and the phosphorylation of Cdk1/Cdc2, are consistent with the ability of SRA to promote MCE of preadipocytes and the terminal differentiation of 3T3-L1 adipocytes.

### SRA regulates inflammatory response genes

Adipocytes are now recognized to form a multifunctional organ not only for lipid storage but also for secreting numerous adipokines and cytokines that play roles in inflammation and insulin sensitivity [1]. Therefore it is interesting that the most enriched GO term associated with the intersection genes is Inflammatory Response, and the fourth such GO term is Regulation of Cytokine Biosynthetic Process (Table 5). As shown in Data Set S3, four intersection genes associated with inflammation were negatively regulated by SRA. These include zinc finger protein 36 (zfp36), toll-like receptor 4 (Tlr4), haptoglobin (Hp) and N-deacetylase/N-sulfotransferase (heparan glucosaminyl) 1 (Ndst1). Perhaps most interesting among these is Tlr4, which can be activated by nutritional fatty acids to promote insulin resistance [49]. The microarray data also identified a number of inflammatory response genes negatively regulated by SRA overexpression in ST2 cells that were not significantly changed in the SRA knockdown cells (Table 9). The most strongly regulated gene in this group is Ccl2 (also known as MCP-1), which is a macrophage-attracting chemokine highly expressed in adipose tissue. Overexpression of MCP-1 has been shown to contribute to macrophage infiltration of adipose tissue and insulin resistance, while ablation of either MCP-1 or its receptor has the opposite effect [50], [51], [52]. The inhibitory effect of SRA on expression of Tlr4, Ccl2 and several additional inflammatory genes was confirmed by RT-qPCR (Figure S3). Overall, these changes suggest that SRA may have anti-inflammatory functions within adipose tissue, which might contribute to the ability of SRA to enhance insulin sensitivity (Figure 5).

**Table 9:** Genes associated with the GO term inflammatory response that have significantly decreased expression in SRA overexpressing ST2 cells.

Probe set	Symbol	Gene name	p-value	Fold change*
**Inflammatory response**				
1420380_at	Ccl2	chemokine (C-C motif) ligand 2	<0.001	−2.84
1421228_at	Ccl7	chemokine (C-C motif) ligand 7	<0.001	−2.36
1416676_at	Kng1	kininogen 1	<0.001	−2.12
1438658_a_at	Edg3	endothelial differentiation, sphingolipid G-protein-coupled receptor, 3	<0.001	−1.72
1425663_at	Il1rn	interleukin 1 receptor antagonist	<0.001	−1.58
1419209_at	Cxcl1	chemokine (C-X-C motif) ligand 1	<0.001	−1.5
1455747_at	Ggtla1	gamma-glutamyltransferase-like activity 1	<0.001	−1.46
1450876_at	Cfh	complement component factor h	<0.001	−1.45
1422758_at	Chst2	carbohydrate sulfotransferase 2	<0.001	−1.15
1425985_s_at	Masp1	mannan-binding lectin serine peptidase 1	<0.001	−1.09
1434376_at	Cd44	CD44 antigen	<0.001	−1.06
1460302_at	Thbs1	thrombospondin 1	0.001	−0.99
1424041_s_at	C1s	complement component 1, s subcomponent	<0.001	−0.95
1417009_at	C1r	complement component 1, r subcomponent	<0.001	−0.93
1450945_at	Prkca	protein kinase C, alpha	<0.001	−0.91
1417268_at	Cd14	CD14 antigen	0.001	−0.66
1449147_at	Chst1	carbohydrate (keratan sulfate Gal-6) sulfotransferase 1	0.002	−0.66
1419132_at	Tlr2	toll-like receptor 2	<0.001	−0.65
1423954_at	C3	complement component 3	<0.001	−0.63
1448460_at	Acvr1	activin A receptor, type 1	0.001	−0.62
1418930_at	Cxcl10	chemokine (C-X-C motif) ligand 10	0.005	−0.56
1449874_at	Ly96	lymphocyte antigen 96	0.001	−0.54
1452985_at	Uaca	uveal autoantigen with coiled-coil domains and ankyrin repeats	0.001	−0.53
1418163_at	Tlr4	toll-like receptor 4	0.003	−0.47
1418099_at	Tnfrsf1b	tumor necrosis factor receptor superfamily, member 1b	0.001	−0.43
1460436_at	Ndst1	N-deacetylase/N-sulfotransferase (heparan glucosaminyl) 1	0.002	−0.39

*Fold change log_2_ format.

### Gene Set Enrichment Analysis (GSEA) identifies functionally related gene sets amongst SRA-responsive genes, including gene sets related to TNFα signaling

**Figure 8:**
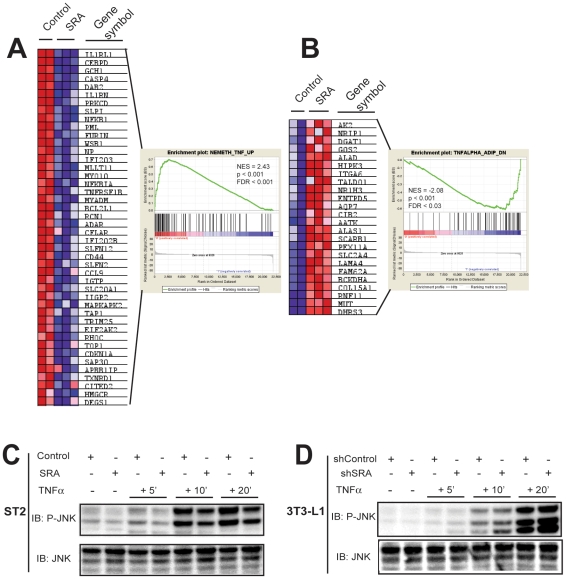
SRA opposes TNFα signaling and inhibits TNFα-induced phosphorylation of JNK. A–B, GSEA was performed on MDIT-differentiated ST2 cells that overexpress SRA versus empty vector control ST2 cells. A, Genes in the gene set NEMETH_TNF_UP are repressed by SRA overexpression. B, Genes in the gene set TNFALPHA_ADIP_DN are induced by SRA overexpression. A and B show the relative expression levels of genes with significantly altered expression displayed as heat maps and as enrichment plots. C and D, SRA inhibits TNFα-induced phosphorylation of JNK. C, Day 4 SRA-overexpresing or control ST2 adipocytes were treated without or with TNFα (20 ng/ml) as indicated. Cell lysates were immunoblotted for phospho-JNK (P-JNK). The membrane was stripped and reprobed for total JNK. D, Similar to C except the cells were day 8 SRA knockdown (shSRA) or control (shControl) 3T3-L1 adipocytes. These results are representative of at least three independent experiments.

GSEA was designed to detect modest but coordinated changes in the expression of groups of functionally related genes [53]. Using GSEA, we identified >100 gene sets that were down-regulated by SRA overexpression in fully differentiated ST2 adipocytes versus control cells (Table S5). Most of these gene sets relate to processes already identified through the GO term analysis: cell cycle, adipocyte differentiation, and cell proliferation. Perhaps most interesting, however, the ninth most enriched gene set to be repressed by SRA overexpression in ST2 cells was NEMETH_TNF_UP (Q<E-06); i.e., SRA repressed genes that are induced by TNFαin this gene set (Figure 8A and Table S5). In contrast, the most significant gene set that was up-regulated by SRA overexpression is TNFALPHA_ADIP_DN; i.e., SRA induced genes that are repressed by TNFα in this gene set (Figure 8B and Table S6). The gene sets enriched by SRA knockdown in 3T3-L1 adipocytes (Tables S7, S8) also overlapped with processes uncovered through the GO term analysis. Interestingly, however, SRA knockdown resulted in up-regulation of the gene set TNFALPHA_30min_UP. These data suggest that SRA may negatively regulate signaling through TNFα, a possibility that we explored subsequently.

### SRA inhibits TNFα phosphorylation of JNK

Proinflammatory cytokines including TNFα are produced in adipose tissue and are secreted from cultured adipocytes *in vitro*. TNFα induces expression of SOCS proteins [54] and activates (phosphorylates) JNK [55]. Both SOCS and JNK are negative regulators of insulin signaling and can cause insulin resistance [56], [57]. Given that the GSEA of our microarray data suggests that SRA may negatively regulate TNFα signaling, we examined the ability of SRA to influence TNFα-induced JNK phosphorylation. As expected, TNFα stimulated JNK phosphorylation in control ST2 adipocytes, but this phosphorylation was impaired in ST2 cells overexpressing SRA (Figure 8C). Conversely, knockdown of SRA in 3T3-L1 adipocytes enhanced TNFα-induced JNK phosphorylation (Figure 8D). These results, combined with the previous data, indicate that SRA has anti-inflammatory effects in adipocytes.

### SRA knockdown in mature adipocytes confirms its ability to enhance insulin sensitivity and inhibit TNFα signaling

**Figure 9:**
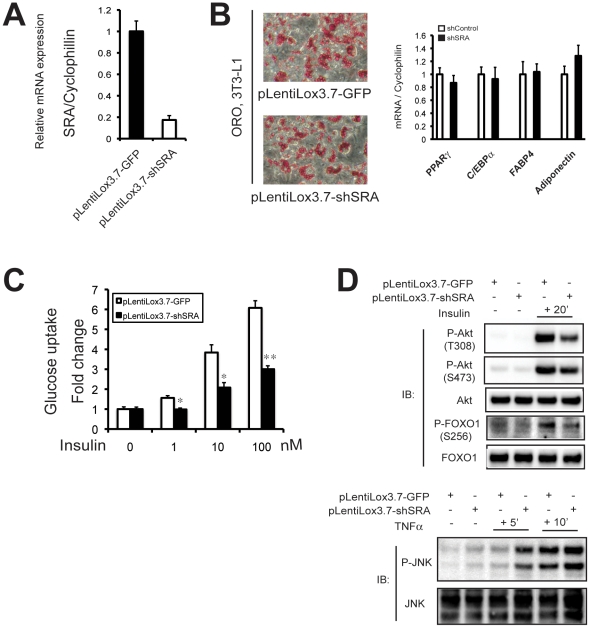
Knockdown of endogenous SRA in mature 3T3-L1 adipocytes confirms that SRA increases insulin action and inhibits TNFα signaling. 3T3-L1 preadipocytes were differentiated under standard hormone cocktail induction (MDI). Day 6 mature adipocytes were infected with lentivirus expressing control (pLentiLox3.7-GFP) or SRA knockdown shRNA (pLentiLox3.7-shSRA), and then were cultured to day 11. A, Efficiency of SRA knockdown was determined by RT-qPCR in day 11 3T3-L1 mature adipocytes. RNA expression was normalized to cyclophilin mRNA. B, Left panel, day 11 control or SRA knockdown adipocytes were stained with Oil Red O to assess lipid accumulation; right panel, mRNA expression for adipocyte marker genes was assessed by RT-qPCR. C, Insulin-stimulated glucose uptake was assayed in day 11 3T3-L1 mature adipocytes, as described in Figure 5. D, Effects of SRA knockdown on insulin dependent phosphorylation of Akt and FOXO1 (upper panel), and TNFα dependent phosphorylation of JNK (lower panel), as described in Figures 5 and 8, respectively. Data are given as the mean ± S.D. Statistical significance was evaluated with Student's t test: * p<0.05 and ** p<0.01. These results are representative of two independent experiments.

In the above studies, we either stably overexpressed SRA in ST2 preadipocytes or knocked down SRA in 3T3-L1 preadipocytes, and then investigated the properties of cells following differentiation. The data indicate that preadipocytes are dependent on SRA for differentiation in response to MDI, but that SRA has very little effect on differentiation by the stronger hormone cocktail MDIT. Since SRA enhances insulin action and represses TNFα signaling in MDIT-differentiated cells (Figures 5, 8), we concluded that these effects likely reflect actions of SRA within the mature adipocyte, rather than effects on adipocyte differentiation. However, to further evaluate this conclusion, we employed a lentiviral system (pLentiLox3.7-shSRA) to stably knock down SRA in already-differentiated 3T3-L1 adipocytes, and then determined the effects of SRA knockdown on insulin and TNFαsignaling. More than 80% of adipocytes were infected by the lentivirus based upon the expression of eGFP encoded by the viral construct (data not shown). As shown in Figure 9A, endogenous SRA was efficiently knocked down in mature 3T3-L1 adipocytes by lentiviral shSRA expression (pLentiLox3.7-shSRA) versus control virus (pLentiLox3.7-GFP). Knockdown of endogenous SRA in mature adipocytes had no effect on the accumulation of neutral lipid, as determined by Oil Red O staining (Figure 9B, left panel), nor on the expression of the adipocyte marker genes PPARγ, C/EBPα, FABP4 or adiponectin (Figure 9B, right panel). Importantly, however, lentiviral SRA knockdown inhibited insulin-stimulated glucose uptake (Figure 9C) to a similar extent as retroviral knockdown in preadipocytes followed by differentiation (Figure 5C). Furthermore, knockdown of SRA in differentiated 3T3-L1 cells inhibited the phosphorylation of Akt and FOXO1 in response to insulin (Figure 9D upper panel), and up regulated TNFα-stimulated JNK phosphorylation (Figure 9D, lower panel). Again, these effects are similar to those observed with retroviral knockdown in preadipocytes, followed by differentiation (Figure 5D). These data suggest SRA may play a direct role in improving insulin sensitivity through up-regulating the insulin signaling pathway and down-regulating TNFα signaling.

## Discussion

The non-coding RNA SRA functions as an RNA coactivator for several nuclear hormone receptors including steroid receptors [26], thyroid hormone receptors [29], [33], retinoic acid receptors [30], vitamin D receptors [33], and steroidogenic factor-1 [34], but it has not been studied in association with PPARγ. SRA is highly expressed in energy-demanding and mitochondria-rich tissues such as skeletal muscle, liver and heart [26]. Here, we show that SRA is expressed at a higher level in mouse WAT than liver. Given that PPARγ is a key transcriptional regulator of adipocyte biology [1], [8], [58], we hypothesized that SRA binds to PPARγ and has functional importance in adipogenesis. Indeed, we found that SRA binds to PPARγ *in vitro*, and that the ligand-binding domain is not required for this interaction. This result is consistent with the binding of SRA to other nuclear hormone receptors. SRA also binds to PPARγ in mature adipocytes, as assessed by RNA-protein co-immunoprecipitation (Figure 2E). Although SRA enhanced PPARγ transactivation in a ligand-dependent manner, it did not increase RXR or C/EBPα mediated transcription, indicating that SRA has some specificity as a coactivator. Our results, together with a recent report that SRA increased PPARδ-dependent reporter gene activity [33], suggest that SRA might be a common coactivator for PPAR family proteins.

The ability of SRA to coactivate PPARγ explains, at least in part, our observations that overexpression of SRA promotes MDI-induced adipogenesis in ST2 cells (Figure 2A–2D), and depletion of SRA impairs MDI-induced adipogenesis in 3T3-L1 cells (Figure 2F–2M). However, SRA has very little effect on adipogenesis in response to the more powerful differentiation cocktail MDIT, presumably because troglitazone allows for strong PPARγ signaling even when SRA levels are low. Although this may seem paradoxical given the observation that SRA functions as a PPARγ coactivator in the presence of the related ligand ciglitazone (Figure 1), those studies were performed in transiently transfected JEG-3 choriocarcinoma cells, which lack the adipocyte milieu.

Gene expression profiling was performed on SRA-overexpressing ST2 adipocytes and SRA knockdown 3T3-L1 adipocytes, versus their respective control adipocytes. The cells had been maximally differentiated with MDIT since, under those conditions, SRA has relatively little effect on adipogenesis. Thus, the microarray analysis should allow for the identification of genes and pathways regulated by SRA in mature adipocytes that may be independent of its action as a PPARγ coactivator to promote adipogenesis. These studies resulted in the identification of 1687 genes with altered expression between SRA overexpresing and control ST2 adipocytes, 340 genes that differed in expression between SRA knockdown and control 3T3-L1 adipocytes, and sixty intersection genes that were oppositely regulated by SRA overexpression and knockdown. Since ST2 cells derive from mouse bone marrow and can differentiate along several different lineages [7], [59], [60], [61], while 3T3-L1 cells are mouse fibroblast preadipocytes [6], it is perhaps not surprising that only a modest overlap is found between the SRA-dependent gene expression changes when these two cell lines are differentiated into adipocytes. We focused our attention primarily on these intersection genes, since they are more likely to be of significance for adipocyte biology. Although many of the changes are quantitatively modest, our functional studies support their biological importance. This most likely reflects the fact that small but coordinated changes in the expression of multiple genes within a metabolic or biochemical pathway can work in concert to have important effects [53]. A GO term analysis revealed over-representation of genes involved in the cell cycle and in inflammatory processes, leading us to study these processes in greater detail.

We identified a novel function of SRA to promote preadipocyte entry into S-phase during mitotic clonal expansion early in adipocyte differentiation, demonstrated both by overexpression in ST2 cells and knockdown in 3T3-L1 cells (Figure 7). Mechanisms may include SRA-induced suppression of p21Cip1 and p27Kip1 expression, and increased phosphorylation of Cdk1/Cdc2 (Figure 7). However, the effect of SRA on p21Cip1 and p27Kip1 expression is biphasic: although SRA decreases expression of these CKIs during MCE, it increases their expression toward late differentiation, when mature adipocytes terminally exit the cell cycle. This suggests that SRA may play a role in exiting from the cell cycle during late differentiation. It has been reported that C/EBPα and PPARγ up-regulate p21Cip1 expression in 3T3-L1 cells [44], [62], and up-regulation of p21Cip1 and p27Kip1 is characteristic of preadipocytes becoming committed to terminal differentiation. Thus, the late effects of SRA to induce expression of these proteins might reflect coactivation of PPARγ.

SRA increases insulin-stimulated glucose uptake and phosphorylation of downstream targets Akt and FOXO1, as shown by overexpression in ST2 adipocytes and knockdown in 3T3-L1 adipocytes (Figures 5). The fact that these effects also are observed following SRA knockdown of already-differentiated 3T3-L1 adipocytes (Figure 9) suggests that they reflect the function of SRA in mature adipocytes, rather than changes in the degree of differentiation. Although C/EBPα is required for insulin-regulated glucose uptake [63], [64], SRA knockdown in already-differentiated adipocytes did not decrease C/EBPα expression (Figure 9B) and SRA does not appear to function as a C/EBPα coactivator (Figure 1E). This suggests that the effect of SRA on glucose uptake is not mediated via C/EBPα. Expression of PPARγ and the PPARγ target gene FABP4 are also not altered by SRA knockdown in mature 3T3-L1 adipocytes (Figure 9B). Thus, SRA may enhance glucose uptake in adipocytes by interaction with other, currently unknown factors. The ability of SRA to regulate expression of genes related to inflammatory processes may be important in its effects on insulin sensitivity, since inflammation causes insulin resistance. For example, the repression of Tlr4, SOCS1 and SOCS3 expression by SRA may contribute to the SRA-induced increase in insulin-stimulated glucose uptake, as might the ability of SRA to induce the expression of Sorbs1. The observation by GSEA that SRA and TNFα regulate a common set of genes in opposite directions further suggests an anti-inflammatory, insulin-sensitizing effect of SRA. This is supported by the finding that SRA overexpression inhibits the ability of TNFα to phosphorylate JNK, whereas SRA knockdown enhances it (Figures 8–9).

Although SRA is a non-coding RNA coactivator for transcription factors [26], [29], [30], [31], [34], expression of a more 5′ extended transcript can lead to production of a protein, SRAP, that also has coactivator function [27], [28]. Our SRA expression vector does not encode SRAP; however, shSRA knockdown would be expected to deplete SRAP protein as well as SRA RNA. Therefore, we cannot rule out that SRA and SRAP together may contribute to the effects on adipocyte biology described herein.

Finally, *in situ* hybridization assays reveal that SRA is predominantly cytoplasmic (∼90%) [65], suggesting that SRA may have extra-nuclear effects. Although we have not explored such putative effects, the ability of SRA to inhibit TNFα-mediated JNK phosphorylation is one such possibility.

In summary, our studies indicate that SRA affects adipogenesis and adipocyte function in multiple ways, including through the coactivation of PPARγ, promotion of S-phase entry during mitotic clonal expansion, and regulation of expression of inflammatory genes and signal transduction in response to insulin and TNFα. Identifying such roles of SRA in adipogenesis and insulin sensitivity thereby provides novel insight into the mechanisms underlying adipocyte physiology and pathophysiology.

## Materials and Methods

### Cell culture, staining and reagents

JEG-3 cells were maintained in minimum essential medium supplemented with 10% FBS with penicillin-streptomycin at 37°C in 5% CO_2_. Mouse 3T3-L1 preadipocytes and human embryonic kidney 293T cells were maintained in Dulbecco's modified Eagle's medium supplemented with 10% calf serum and penicillin-streptomycin at 37°C in 10% CO_2_. Mouse marrow-derived ST2 cells were incubated at 37°C in 5% CO_2_ in α-minimal essential medium supplemented with 10% FBS and penicillin-streptomycin. Induction of 3T3-L1 or ST2 cell differentiation was achieved by treatment of 2 day post-confluent cells (day 0) in media supplemented with 10% FBS and a hormone cocktail containing 3-isobutyl-1-methylxanthine (0.5 mM), dexamethasone (1 µM), and insulin (0.167 µM), denoted MDI. On day 2, the cells were treated again with 0.167 µM insulin, and subsequently were refed with growth media containing 10% FBS every 2 days. In some studies, troglitazone (50 mM in dimethylsulfoxide) was added to the hormone cocktail to achieve a final media concentration of 5 µM (MDIT). Lipid accumulation in adipocytes was visualized by staining with Oil Red O. Briefly, differentiated adipocytes were washed with phosphate buffered saline (PBS) and fixed with 10% formaldehyde in PBS for 4 min. After two washes with water, cells were stained for 1–2 hrs with Oil Red O working solution (0.3% (wt/vol) in 60% isopropanol). The stain was aspirated and cells were washed at least twice with water and photographed.

Recombinant mouse TNFα was purchased from R&D Systems, Inc. (Minneapolis, MN) and was reconstituted in PBS containing 0.1% bovine serum albumin (BSA). Antibodies against the following proteins were obtained as indicated: PPARγ (H-100, sc-7196 and E-8, sc-7273), C/EBPα (14AA, sc-61) and GAPDH (6C5, sc-32233) from Santa Cruz Biotechnology (Santa Cruz, CA); FABP4 (Cat# MAB1443) from R&D Systems, Inc.; p21Cip1 (Cat# 556430), p27Kip1 (Cat# 610241), pY15-Cdk1/Cdc2 (Cat# 612306) and Cdk1/Cdc2 (Cat# 610037) from BD Transduction Laboratories (San Jose, CA); phospho-Akt (Ser473) (Cat# 9271), phospho-Akt (Thr308) (Cat# 9275), Akt (Cat# 9272), phospho-FOXO1 (Ser256) (Cat# 9461), FOXO1 (C29H4) (Cat# 2880), phospho-SAPK/JNK (Thr183/Tyr185) (81E11) and SAPK/JNK (56G8) from Cell Signaling Technology (Danvers, MA), and adiponectin (Cat# A6354) from Sigma (Saint Louis, MO).

### Plasmids, transfection, retroviral infection and reporter gene assays

The vector pGEX-KG [66] was used to express human PPARγ1 protein as a glutathione S-transferase (GST) fusion in *Escherichia coli*. To maximize the recovery of full-length protein, the construct was further tagged with six histidines at its C-terminus. For simplicity, we refer to this protein as GST-PPARγ. Deletion mutants of GST-PPARγ were constructed by inverse PCR and are depicted in Figure 1A. PPARγ was expressed in mammalian cells from the vector pFlag-CMV-7.1 (Sigma). Mouse RXRα was expressed from the vector pCDM. The reporter plasmid pPPRE-luc (a gift from Amy Au, Kolling Institute of Medical Research, St. Leonards, Australia) contains three copies of the acyl-CoA oxidase PPAR response element upstream of TK-luciferase [67]. The human SRA isoform 2 expression vector pSCT-SRA was kindly provided by R. Lanz (Baylor College of Medicine, Houston, TX). The leptin promoter-luciferase reporter plasmid pObluc-760 (a gift from M. Daniel Lane, John Hopkins University, Baltimore, MD) and the p42 C/EBPα expression plasmid were as previously described [68]. To create the retroviral expression vector pMSCV-SRA, pSCT-SRA was linearized with EcoRI and partially digested with BamHI. The SRA cDNA was gel purified and ligated to pMSCV [69] that had been digested with BglII and EcoRI.

Transient transfections and luciferase reporter gene assays were performed as described previously [34]. For retroviral infection, 293T cells were transfected by calcium phosphate coprecipitation with pMSCV or pMSCV-SRA retroviral expression vectors and viral packing vectors SVε-E-MLV-env and SVψ-E-MLV as described previously [39]. Virus-containing media were collected 16 h after transfection and passed through a 0.45-um syringe filter. Polybrene was added to a final concentration of 8 µg/ml. The media were then applied to subconfluent (40%) ST2 cells in 10-cm plates. The infection protocol was repeated every 8–16 h until the cells were 80% confluent. The cells were then trypsin-treated and replated in α-minimal essential medium supplemented with 10% FBS, penicillin-streptomycin and 3 µg/ml puromycin for selection.

### GST fusion protein purification and in vitro RNA binding assay

The purification of GST- PPARγ and mutant versions from *E. coli* strain BL21 by sequential cobalt and glutathione agarose column chromatography, as well as the *in vitro* RNA binding assay, were performed essentially as described [29]. The ^32^P-labeled RNA SRA probe was made by *in vitro* transcription using T7 RNA polymerase and [α-^32^P]UTP from pSCT-SRA that had been linearized by digestion with PvuII.

### Gene silencing by short hairpin RNA (shRNA)

Knockdown of endogenous SRA in 3T3-L1 preadipocytes was performed by infection with a retrovirus expressing either of two shRNAs targeting SRA. Two different 21-nt shRNA constructs targeting mouse SRA1 mRNA were designed using Invitrogen's web design. The sense oligonucleotides were: shSRA-1 5′ GGCACTGCTAGTGCAAGAACT 3′, and shSRA-2 5′ GACCACTGCAAGATCACTTGT 3′. The oligonucleotides were cloned into the retroviral pSUPERIOR.retro.puro vector (OligoEngine, Seattle, WA) with a sense-loop-antisense design, using the loop sequence CTTCCTGTCA. The sense oligonucleotide included a BglII-compatible overhang at its 5′ end, and the antisense oligonucleotide similarly contained a HindIII-compatible overhang. The oligonucleotides (250 pmol each) were annealed in 10 mM Tris (pH 7.5) and 100 mM NaCl (50 µl) with conditions of 95°C for 3 min, then 25 cycles of 20 seconds with a 1°C decrement per cycle, 70°C for 10 minutes and then 65 cycles of 20 seconds with a 1°C decrement per cycle, then 4°C. Annealed oligonucleotides were ligated into the pSUPERIOR.retro.puro vector that had been digested with BglII and HindIII and gel purified. Ligation was performed using 1 µl of the annealed oligonucleotide mix, 75 ng of digested vector, T_4_ DNA ligase and 1X ligation buffer in a 10 µl reaction. Ligation was performed at conditions of 100 cycles of 22°C for 20 seconds and 12°C for 1 minute. The ligation mix was transformed into competent *E. coli* DH5α. Purified plasmids were sequenced to verify the correct insert. The non-targeting shRNA control was as described [34].

Endogenous SRA in 3T3-L1 mature adipocytes was knocked down by infection with a lentivirus expressing shRNA directed against SRA. Briefly, the vector pLentiLox3.7-shSRA that expresses shRNA-2 for SRA (targeting sequence as described above) was constructed by inserting pairs of annealed DNA oligonucleotides into the pLentiLox3.7-GFP vector between the Hpal and Xho1 restriction sites [70]. 293T cells were cotransfected with either shRNA (pLentiLox3.7-shSRA) or control (pLentiLox3.7-GFP) plasmids and the packaging plasmids psPAX2 and pMD2.G by the calcium phosphate method to produce the lentivirus. The virus-containing media were collected, passed through 0.45 µm filters, supplemented with 8 µg/ml Polybrene (Sigma) and used to infect day 6 mature 3T3-L1 adipocytes that had been differentiated with the hormone cocktail MDI at day 0. The 293T cell media were replaced with fresh media, and were subsequently used to repeat the 3T3-L1 cell infection two additional times at intervals of 8 to 12 h. The shSRA or control lentivirus infected adipocytes were cultured until day 11, at which time experiments were performed to evaluate insulin stimulated glucose uptake and signal transduction in response to insulin and TNFα.

### Glucose uptake assay

Day 4 ST2 or day 8 to 11 3T3-L1 adipocytes were incubated for 3 h in serum- and glucose-free media. Basal and insulin-induced glucose uptakes were measured by rinsing the cells and then incubating in serum- and glucose-free media for 30 min with or without insulin, followed by the addition of unlabeled 2-deoxy-D-glucose (920 µM) and 1 µCi of 2-deoxy-D-[^14^C] glucose (Amersham Biosciencess/GE Healthcare). After incubation for 5 min at room temperature, the media were removed, and the cells were washed in cold PBS and lysed in 750 µl of 0.1% SDS. The radioactivity associated with 250 µl was determined by liquid scintillation counting. The results were corrected for nonspecific binding using control cells incubated in the presence of 20 µM cytochalasin B. Glucose uptake was normalized to protein content as measured from the remaining cell lysate with Bio-Rad protein reagent.

### Cell lysis, immunoblotting and RNA-protein immunoprecipitations

Cells were lysed in buffer containing 40 mM HEPES, 120 nM sodium chloride, 10 mM sodium pyrophosphate, 10 mM sodium glycerophosphate, 1 mM EDTA, 50 mM sodium fluoride, 0.5 mM sodium orthovanadate, and 1% Triton X-100. Cell lysates were gently resuspended by triteration and incubating at 4°C with gentle rocking for 40 min to 1 h, followed by microcentrifugation for 10 min at 4°C. The supernatants were transferred to new tubes and protein concentrations were determined. Proteins were separated by SDS-PAGE and transferred onto polyvinylidene difluoride membranes, and immunoblotting was performed using the antibodies described above. Detection was with a SuperSignal West Dura kit (Thermo Fisher Scientific, Rockford, IL) and a Bio-Rad Fluor-S Max Multi-Imager.

To test whether SRA binds to PPARγ in adipocytes, an RNA-protein immunoprecipitation was performed as described previously [34] with minor modifications. Briefly, day 8 3T3-L1 adipocytes were washed twice with PBS, then chemical cross-linking of RNA and protein was performed by addition of 0.1% formaldehyde for 10 minutes. After the addition of 0.25 M glycine, the cells were harvested and lysed in RIPA buffer containing complete protease inhibitor cocktail (Roche, Indianapolis, IN) and RNase inhibitor (40 U/µl RNasin [Promega, Madison, WI]) and followed by sonication. After centrifugation, the lysate supernatants were treated with DNase I (Ambion, Austin, TX) for 30 min at 37°C, and then 20 mM EDTA was added to stop the enzyme activity. Next, cell lysates were diluted 1∶10 with RIPA buffer containing nonspecific yeast tRNA (100 µg/ml) and were precleared by incubation with protein A beads (Millipore, Billerica, MA) for 30 min to 1 h at 4°C. For immunoprecipitation, 3 µg of either normal IgG or anti-PPARγ (H-100 or E-8) were added to the precleared supernatants and incubated at 4°C overnight with gentle rocking. The lysates were then incubated with 50 µl protein A beads containing 5% BSA, protease inhibitor cocktail and RNasin for 2–3 h at 4°C. The beads were pelleted, washed 5 times with high-stringency RIPA buffer, and the cross-links were reversed for 45 min at 70°C in reversal buffer as described [34]. RNA was finally isolated by phenol-chloroform-isoamyl alcohol extraction and ethanol precipitation containing glycogen (Ambion), and was used for first-strand cDNA synthesis with SuperScript III (Invitrogen, Carlsbad, CA) and random hexamer primers. Real-time PCR was done using 2 µl of the resulting cDNA and primers that amplify mouse SRA. Data were normalized to the signal obtained using primers that amplify cyclophilin.

### Cell cycle analysis and BrdU labeling

Growth-arrested ST2 or 3T3-L1 preadipocytes, before or every 2 hr after induction with hormone cocktail MDIT, were harvested by trypsinization, washed twice in PBS and fixed with cold 50% EtOH for a minimum of 20 min. Fixed cells were washed with PBS and incubated in the dark for at least 30 min with 50 µg/ml propidium iodide (PI) and 100 µg/ml RNaseA in PBS. Labeled cells were analyzed using a FACSCalibur flow cytometry system (BD Biosciences, San Jose, CA) and cell cycle profiles were determined using ModFit software (Becton Dickinson, San Diego, CA).

For BrdU labeling, SRA overexpressing or control ST2 preadipocytes were plated on chamber coverglass slides and maintained in the ST2 cell media described above until one day after confluence was achieved. Cells were induced to differentiate with MDIT, and 10 h after induction were labeled for 4 h with 20 µM BrdU. The cells were fixed for 1 min in fresh 1∶1 methanol:acetone, blocked 1 h at room temperature in blocking buffer [34], incubated with anti-BrdU mouse monoclonal primary antibody (1∶500) (Calbiochem, La Jolla, CA, Cat# NA61) in blocking buffer overnight at 4°C, and then incubated with FITC-conjugated goat anti-mouse IgG (H+L) at 1∶200 (AnaSpec, Inc., San Jose, CA) in blocking buffer for 2 h at room temperature. The cell nuclei were stained with 1∶20,000 Hoechst 33342 for 5 min at room temperature. After each step, the cells were washed three times with PBS. Finally, anti-fade reagent (ProLong Gold, Invitrogen) was added to the monolayers and coverslips were mounted on the slides. Microscopy was carried out using an Olympus BX-51A fluorescent microscope at a magnification of ×40. The percentage of BrdU positive cells was calculated by comparing the number of BrdU labeled cells with the total number of cells using ImageJ software [71].

### Affymetrix microarray analysis

Gene expression profiling was performed to compare SRA overexpressing (pMSCV-SRA) versus control (pMSCV vector) ST2 adipocytes (day 4), or endogenous SRA knockdown (pSuperior-shSRA-2) and control (pSuperior-shcontrol) 3T3-L1 adipocytes (day 8). Total RNA was extracted from the above fully (MDIT) differentiated adipocytes with Trizol reagent (Invitrogen) and cleaned up by RNeasy spin column (Qiagen Cat# 74104). Biotin-labeled cRNAs were prepared according to the Affymetrix GeneChip Expression Analysis Technical Manual. Hybridizations were performed on GeneChip mouse Genome 430 2.0 arrays (Affymetrix). To check the overall quality of the raw data, several quality control plots were made before the statistical analysis for significant changes of gene expression. One plot showed the distribution of perfect match probe intensities to confirm their consistency for each chip and the other looked at similarities in the RNA degradation between samples to help ensure differences in gene expression could not be attributed to differences in degradation. The expression values (log_2_ transformed data) were then calculated using a robust multi-array average (RMA) [72]. This is a modeling strategy that converts the perfect match probe value into an expression value for each gene. As a final quality control step we performed a principle component analysis and plotted the first two principle components to show that replicated samples were grouping together. The microarray data have been deposited in the Gene Expression Omnibus (accession number GSE21594).

Prior to making comparisons, we filtered probsets based on a variance over all samples of 0.05. This removed those probesets that were unchanged in any sample, which were by definition not interesting to us. After filtering there were 19068 of 45101 probesets that remained.

We then fitted a linear model to the data that was specifically designed for microarray analysis [73] using the limma package of Bioconductor in the R statistical environment. This was almost identical to fitting a *t*-statistic to each probeset, but by first fitting a linear model we were able to determine if a particular comparison was significant while still accounting for the significance of the same comparison in the other cells line (e.g. testing if ST2 SRA overexpression vs. control is significant when 3T3-L1 SRA knockdown vs. control is already known to be significant). This modeling strategy also helped us to increase power to detect differences in two ways. First, the denominator of the *t*-statistic, which estimates the variance of the comparison, is known to be less accurate when there is not much replication. Here we used an overall variance estimate from all probesets to increase the accuracy of the observed variance for each comparison. Second, the denominator of a contrast was based on the variance of all the samples rather than just those under consideration. This increased the number of samples used to compute the variance, which tended to increase our power to make correct decisions. Furthermore, we selected probesets based on an adjusted p-value of 0.05, adjusted using Benjamini Hochberg false discovery rate (FDR) [74].

Gene ontology (GO) analysis was performed for the genes (probesets) with significant changes (p<0.05). The GOstats package of Bioconductor was used to test the association between Gene Ontology terms and the gene list [75]. This was done using a conditional hypergeometric test; conditioning means that each term was tested for statistical overrepresentation after removing genes that overlapped a significant child term. The gene universe was defined as all genes on the array after a nonspecific filter removed genes with a variance over all samples less than .05.

Gene set enrichment analysis (GSEA) was performed as described [53]. All of the gene sets examined were obtained from the Molecular Signature Database C2, which includes 1892 metabolic and signaling pathways derived from 12 publicly available and manually curated databases.

To confirm expression level changes of mRNAs altered by either SRA overexpression or knockdown, reverse transcription-real time PCR (RT-qPCR) was performed. Briefly, total RNA from ST2 or 3T3-L1 adipocytes was isolated with Trizol reagent. Four µg of total RNA were reverse transcribed using SuperScript III and qPCR was performed as described previously [34]. Primer sequences for each SRA responsive gene used for RT-qPCR analysis are provided in Table S9.

## Supporting Information

Figure S1 SRA effects on adipocyte differentiation and gene expression. A, Control (pMSCV empty vector; Cont.) or SRA overexpressing ST2 cells were grown to confluence and treated with FBS, MDI or MDIT to induce adipocyte differentiation. On day 4 of differentiation, cells were stained with Oil Red O to identify triacylglycerol droplets. B, 3T3-L1 preadipocytes were infected with retroviruses expressing either a control short hairpin RNA (shControl) or one of two shRNAs directed against mouse SRA (shSRA-1 or -2). Preadipocytes were induced to differentiate with MDI or MDIT. At day 8, adipocytes were stained with Oil Red O. C, Control and SRA overexpressing ST2 adipocytes differentiated by MDIT were analyzed at day 4 by RT-qPCR for the expression of C/EBPα, PPARγ, FABP4 and adiponectin, normalized to cyclophilin. D, shControl and shSRA-2 3T3-L1 adipocytes differentiated by MDIT were analyzed at day 8 by RT-qPCR for the expression of C/EBPα, PPARγ, FABP4 and adiponectin, normalized to cyclophilin. E, Whole cell extracts from shControl and shSRA-2 3T3-L1 adipocytes differentiated by MDIT at day 8 were subjected to immunoblotting for PPARγ or C/EBPα. The membranes were stripped and reprobed for GAPDH as a loading control. These results are representative of at least three independent experiments.
(2.70 MB TIF)

Figure S2 Confirmation of SRA effect on S-phase entry during preadipocyte mitotic clonal expansion by BrdU incorperation. A, Immunocytochemistry demonstrates an increase in BrdU positive SRA overexpressing ST2 preadipocytes, versus empty vector control ST2 preadipocytes. The cells were exposed to BrdU 10–14 hours post-induction by MDIT. B, Quantification of the data from B, showing a significant increase in fraction of BrdU positive cells in SRA overexpressing ST2 preadipocytes, confirming an increase in S-phase cells (2500 nuclei from random fields were counted per construct); **p < 0.01 by Student's t-test. Experiments were repeated at least three times with triplicate samples.
(1.35 MB TIF)

Figure S3 RT-qPCR analyses confirm expression changes of SRA responsive inflammatory genes in ST2 adipocytes. Total RNA was isolated from day 4 MDIT-differentiated SRA overexpressing or empty vector control ST2 cells. Gene expression was then analyzed by RT-qPCR, normalized to cyclophilin. Significance was determined by Student's t-test compared to each control. * P < 0.05, **P < 0.01 and *** p < 0.001. Experiments were repeated at least three times with triplicate samples.
(0.28 MB TIF)

Table S1 GO terms in biological processes (BP) overrepresented amongst genes with altered expression in SRA overexpressing versus empty vector control ST2 adipocytes.
(0.08 MB DOC)

Table S2 GO terms in molecular function (MF) overrepresented amongst genes with altered expression in SRA overexpressing versus empty vector control ST2 adipocytes.
(0.05 MB DOC)

Table S3 GO terms in biological processes (BP) overrepresented amongst genes with altered expression in endogenous SRA knockdown versus control 3T3-L1 adipocytes.
(0.05 MB DOC)

Table S4 GO terms in molecular function (MF) overrepresented amongst genes with altered expression in endogenous SRA knockdown versus control 3T3-L1 adipocytes.
(0.04 MB DOC)

Table S5 Down-regulated gene sets by SRA overexpression in ST2 cells analyzed by GSEA
(0.22 MB DOC)

Table S6 Up-regulated gene sets by SRA overexpression in ST2 cells analyzed by GSEA.
(0.04 MB DOC)

Table S7 Down-regulated gene sets by SRA knockdown in 3T3-L1 cells analyzed by GSEA.
(0.05 MB DOC)

Table S8 Up-regulated gene sets by shSRA knockdown in 3T3-L1 cells analyzed by GSEA.
(0.21 MB DOC)

Table S9 Primers used in real time PCR.
(0.05 MB DOC)

Data Set S1 (0.97 MB XLS)

Data Set S2 (0.08 MB XLS)

Data Set S3 (0.02 MB XLS)
