# Supplementary figures and images for: Multiple Roles for the Non-Coding RNA SRA in Regulation of Adipogenesis and Insulin Sensitivity
Source: PLoS One. 2010 Dec 2;5(12):e14199. doi: 10.1371/journal.pone.0014199 (PMC2996286; doi:10.1371/journal.pone.0014199)

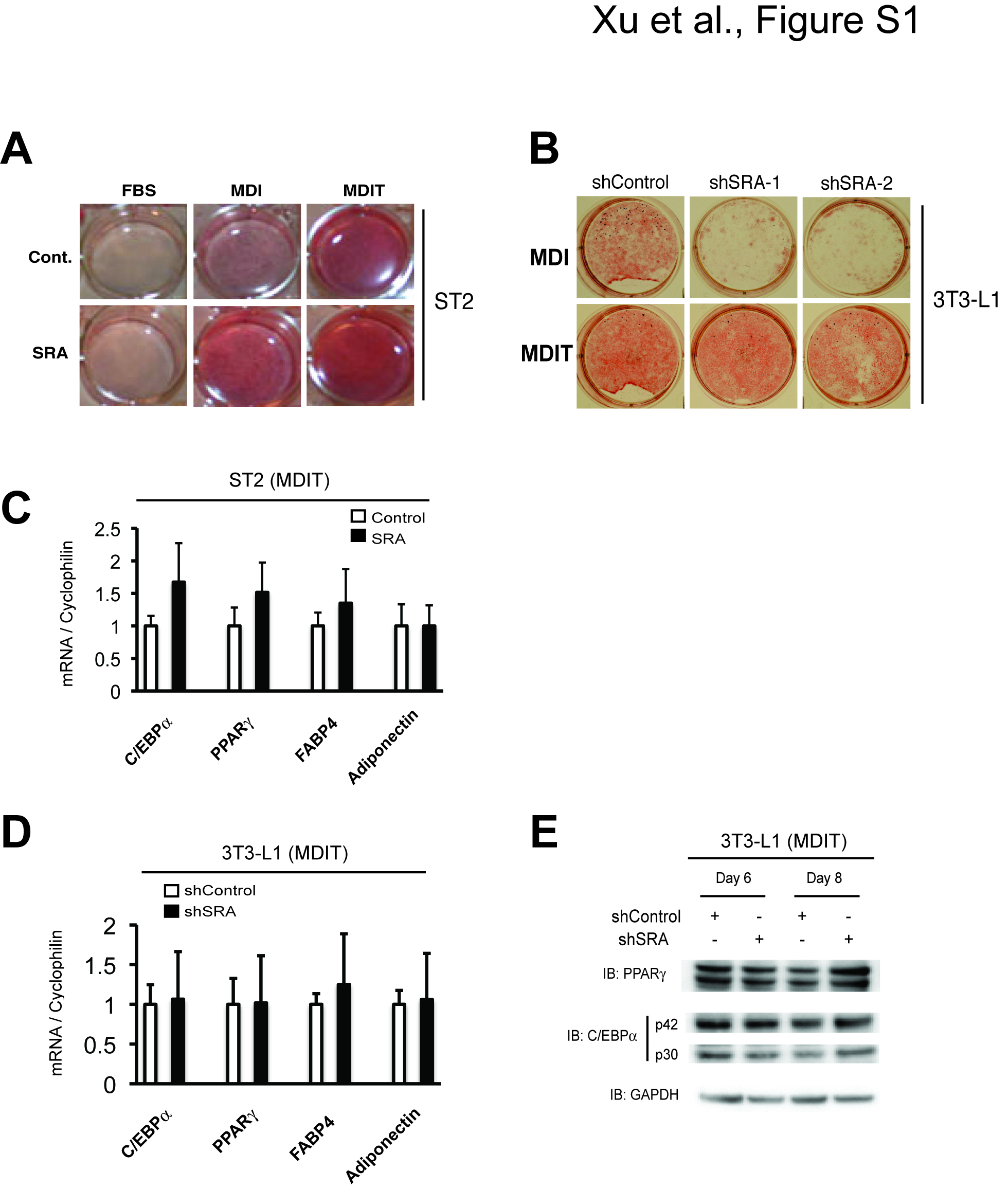

Supplement: Figure S1 — SRA effects on adipocyte differentiation and gene expression. A, Control (pMSCV empty vector; Cont.) or SRA overexpressing ST2 cells were grown to confluence and treated with FBS, MDI or MDIT to induce adipocyte differentiation. On day 4 of differentiation, cells were stained with Oil Red O to identify triacylglycerol droplets. B, 3T3-L1 preadipocytes were infected with retroviruses expressing either a control short hairpin RNA (shControl) or one of two shRNAs directed against mouse SRA (shSRA-1 or -2). Preadipocytes were induced to differentiate with MDI or MDIT. At day 8, adipocytes were stained with Oil Red O. C, Control and SRA overexpressing ST2 adipocytes differentiated by MDIT were analyzed at day 4 by RT-qPCR for the expression of C/EBPα, PPARγ, FABP4 and adiponectin, normalized to cyclophilin. D, shControl and shSRA-2 3T3-L1 adipocytes differentiated by MDIT were analyzed at day 8 by RT-qPCR for the expression of C/EBPα, PPARγ, FABP4 and adiponectin, normalized to cyclophilin. E, Whole cell extracts from shControl and shSRA-2 3T3-L1 adipocytes differentiated by MDIT at day 8 were subjected to immunoblotting for PPARγ or C/EBPα. The membranes were stripped and reprobed for GAPDH as a loading control. These results are representative of at least three independent experiments. (2.70 MB TIF) [file pone.0014199.s001.tif]

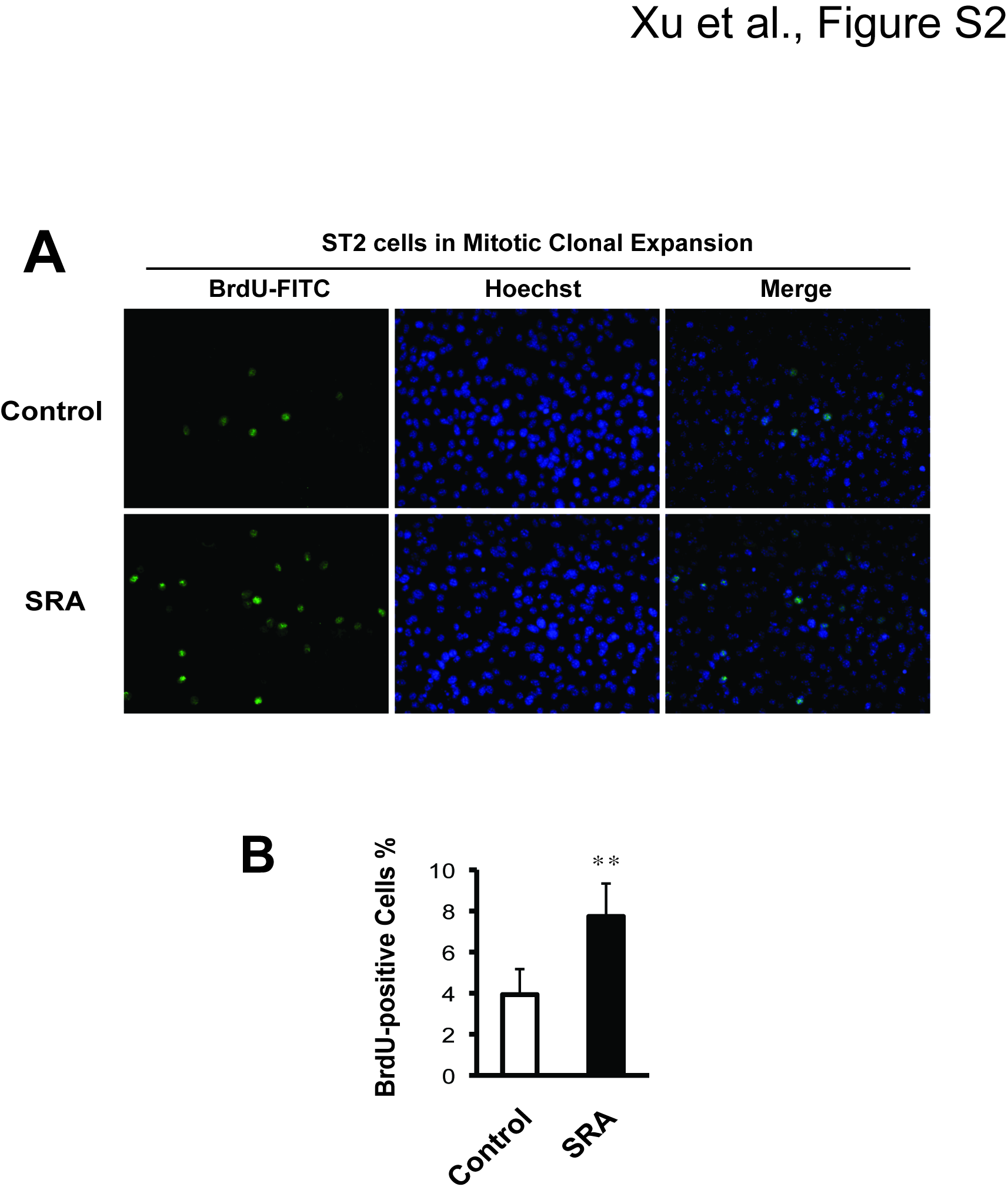

Supplement: Figure S2 — Confirmation of SRA effect on S-phase entry during preadipocyte mitotic clonal expansion by BrdU incorperation. A, Immunocytochemistry demonstrates an increase in BrdU positive SRA overexpressing ST2 preadipocytes, versus empty vector control ST2 preadipocytes. The cells were exposed to BrdU 10–14 hours post-induction by MDIT. B, Quantification of the data from B, showing a significant increase in fraction of BrdU positive cells in SRA overexpressing ST2 preadipocytes, confirming an increase in S-phase cells (2500 nuclei from random fields were counted per construct); **p < 0.01 by Student's t-test. Experiments were repeated at least three times with triplicate samples. (1.35 MB TIF) [file pone.0014199.s002.tif]

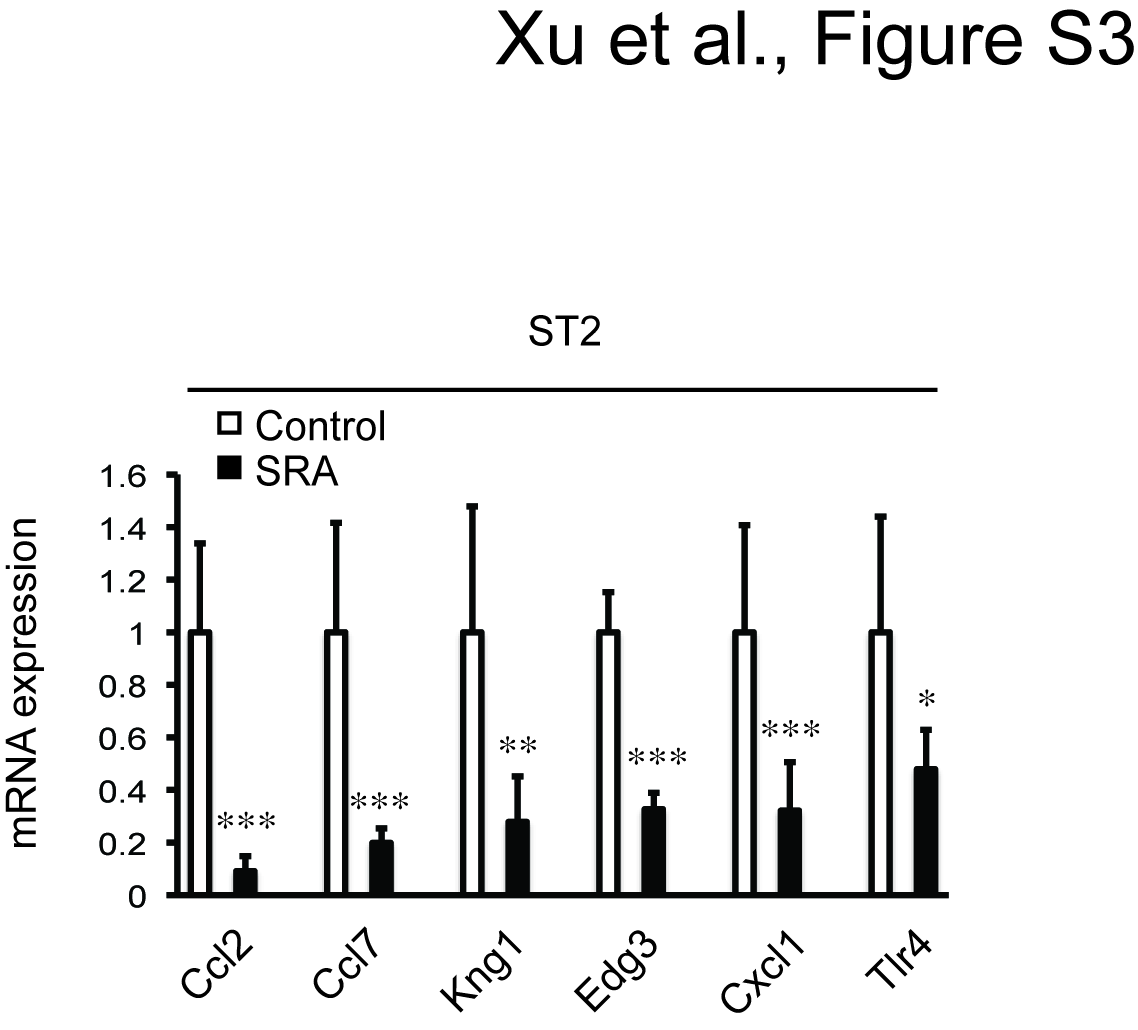

Supplement: Figure S3 — RT-qPCR analyses confirm expression changes of SRA responsive inflammatory genes in ST2 adipocytes. Total RNA was isolated from day 4 MDIT-differentiated SRA overexpressing or empty vector control ST2 cells. Gene expression was then analyzed by RT-qPCR, normalized to cyclophilin. Significance was determined by Student's t-test compared to each control. * P < 0.05, **P < 0.01 and *** p < 0.001. Experiments were repeated at least three times with triplicate samples. (0.28 MB TIF) [file pone.0014199.s003.tif]
