# Supplementary material for: Multiple Roles for the Non-Coding RNA SRA in Regulation of Adipogenesis and Insulin Sensitivity
Source: PLoS One. 2010 Dec 2;5(12):e14199. doi: 10.1371/journal.pone.0014199 (PMC2996286; doi:10.1371/journal.pone.0014199)
Supplement: Table S1 — GO terms in biological processes (BP) overrepresented amongst genes with altered expression in SRA overexpressing versus empty vector control ST2 adipocytes. (0.08 MB DOC) [file pone.0014199.s004.doc]

**Table S1.** GO terms in biological processes (BP) overrepresented amongst genes with altered expression in SRA overexpressing versus empty vector control ST2 adipocytes.

| GO BP ID | Pvalue | ExpCount | Count | Size | Term |
| --- | --- | --- | --- | --- | --- |
| GO:0000278 | 0 | 26 | 58 | 153 | mitotic cell cycle |
| GO:0007067 | 0 | 20 | 46 | 115 | mitosis |
| GO:0006955 | 0 | 32 | 64 | 188 | immune response |
| GO:0007155 | 0 | 47 | 85 | 278 | cell adhesion |
| GO:0051301 | 0 | 29 | 57 | 169 | cell division |
| GO:0000279 | 0 | 25 | 51 | 146 | M phase |
| GO:0016477 | 0 | 27 | 46 | 156 | cell migration |
| GO:0051674 | 0 | 31 | 51 | 181 | localization of cell |
| GO:0000074 | 0 | 38 | 60 | 223 | regulation of progression through cell cycle |
| GO:0001944 | 0 | 22 | 39 | 129 | vasculature development |
| GO:0006260 | 0 | 16 | 31 | 96 | DNA replication |
| GO:0048731 | 0 | 144 | 181 | 847 | system development |
| GO:0006817 | 0 | 5 | 13 | 28 | phosphate transport |
| GO:0042127 | 0.001 | 33 | 51 | 193 | regulation of cell proliferation |
| GO:0009605 | 0.001 | 33 | 51 | 195 | response to external stimulus |
| GO:0007229 | 0.001 | 7 | 16 | 43 | integrin-mediated signaling pathway |
| GO:0006954 | 0.002 | 15 | 27 | 90 | inflammatory response |
| GO:0001525 | 0.002 | 15 | 27 | 90 | angiogenesis |
| GO:0007165 | 0.002 | 200 | 235 | 1174 | signal transduction |
| GO:0009653 | 0.002 | 102 | 128 | 599 | anatomical structure morphogenesis |
| GO:0045664 | 0.002 | 3 | 8 | 16 | regulation of neuron differentiation |
| GO:0048699 | 0.003 | 28 | 42 | 164 | generation of neurons |
| GO:0001657 | 0.003 | 3 | 9 | 20 | ureteric bud development |
| GO:0032501 | 0.003 | 199 | 232 | 1185 | multicellular organismal process |
| GO:0001822 | 0.003 | 7 | 14 | 39 | kidney development |
| GO:0045444 | 0.004 | 3 | 8 | 17 | fat cell differentiation |
| GO:0007169 | 0.004 | 13 | 22 | 74 | transmembrane receptor protein tyrosine kinase signaling pathway |
| GO:0006952 | 0.005 | 13 | 22 | 75 | defense response |
| GO:0048522 | 0.005 | 68 | 88 | 401 | positive regulation of cellular process |
| GO:0006270 | 0.005 | 2 | 6 | 11 | DNA replication initiation |
| GO:0007010 | 0.005 | 43 | 59 | 253 | cytoskeleton organization and biogenesis |
| GO:0007059 | 0.006 | 5 | 11 | 29 | chromosome segregation |
| GO:0006935 | 0.006 | 7 | 14 | 41 | chemotaxis |
| GO:0051239 | 0.006 | 22 | 34 | 131 | regulation of multicellular organismal process |
| GO:0008286 | 0.006 | 3 | 8 | 18 | insulin receptor signaling pathway |
| GO:0022403 | 0.006 | 4 | 10 | 26 | cell cycle phase |
| GO:0007160 | 0.008 | 6 | 12 | 34 | cell-matrix adhesion |
| GO:0030199 | 0.009 | 2 | 6 | 12 | collagen fibril organization |
| GO:0035295 | 0.009 | 17 | 27 | 101 | tube development |
| GO:0048514 | 0.009 | 4 | 9 | 23 | blood vessel morphogenesis |
| GO:0048754 | 0.01 | 7 | 13 | 39 | branching morphogenesis of a tube |
