# Supplementary material for: Multiple Roles for the Non-Coding RNA SRA in Regulation of Adipogenesis and Insulin Sensitivity
Source: PLoS One. 2010 Dec 2;5(12):e14199. doi: 10.1371/journal.pone.0014199 (PMC2996286; doi:10.1371/journal.pone.0014199)
Supplement: Table S2 — GO terms in molecular function (MF) overrepresented amongst genes with altered expression in SRA overexpressing versus empty vector control ST2 adipocytes. (0.05 MB DOC) [file pone.0014199.s005.doc]

**Table S2.** GO terms in molecular function (MF) overrepresented amongst genes with altered expression in

SRA overexpressing versus empty vector control ST2 adipocytes.

| GO MF ID | Pvalue | ExpCount | Count | Size | Term |
| --- | --- | --- | --- | --- | --- |
| GO:0005509 | 0 | 65 | 102 | 393 | calcium ion binding |
| GO:0004872 | 0 | 56 | 85 | 339 | receptor activity |
| GO:0003779 | 0 | 23 | 41 | 138 | actin binding |
| GO:0060089 | 0 | 115 | 151 | 691 | molecular transducer activity |
| GO:0030020 | 0 | 3 | 10 | 18 | extracellular matrix structural constituent conferring tensile strength |
| GO:0003924 | 0.001 | 16 | 29 | 95 | GTPase activity |
| GO:0016538 | 0.001 | 3 | 9 | 17 | cyclin-dependent protein kinase regulator activity |
| GO:0017048 | 0.001 | 2 | 8 | 14 | Rho GTPase binding |
| GO:0001871 | 0.001 | 8 | 18 | 51 | pattern binding |
| GO:0030246 | 0.001 | 20 | 34 | 121 | carbohydrate binding |
| GO:0005543 | 0.002 | 17 | 29 | 101 | phospholipid binding |
| GO:0005539 | 0.002 | 8 | 17 | 49 | glycosaminoglycan binding |
| GO:0005515 | 0.003 | 420 | 463 | 2538 | protein binding |
| GO:0004222 | 0.003 | 8 | 16 | 47 | metalloendopeptidase activity |
| GO:0003774 | 0.003 | 11 | 20 | 65 | motor activity |
| GO:0004182 | 0.004 | 2 | 7 | 14 | carboxypeptidase A activity |
| GO:0019838 | 0.005 | 5 | 12 | 33 | growth factor binding |
| GO:0004857 | 0.005 | 15 | 25 | 90 | enzyme inhibitor activity |
| GO:0016462 | 0.006 | 51 | 68 | 306 | pyrophosphatase activity |
| GO:0004888 | 0.006 | 41 | 57 | 249 | transmembrane receptor activity |
| GO:0008009 | 0.006 | 2 | 7 | 15 | chemokine activity |
| GO:0048503 | 0.007 | 8 | 16 | 51 | GPI anchor binding |
| GO:0016817 | 0.007 | 51 | 68 | 308 | hydrolase activity, acting on acid anhydrides |
| GO:0004180 | 0.008 | 4 | 9 | 23 | carboxypeptidase activity |
| GO:0008083 | 0.01 | 12 | 20 | 71 | growth factor activity |
