# Supplementary material for: Multiple Roles for the Non-Coding RNA SRA in Regulation of Adipogenesis and Insulin Sensitivity
Source: PLoS One. 2010 Dec 2;5(12):e14199. doi: 10.1371/journal.pone.0014199 (PMC2996286; doi:10.1371/journal.pone.0014199)
Supplement: Table S3 — GO terms in biological processes (BP) overrepresented amongst genes with altered expression in endogenous SRA knockdown versus control 3T3-L1 adipocytes. (0.05 MB DOC) [file pone.0014199.s006.doc]

**Table S3.** GO terms in biological processes (BP) overrepresented amongst genes with altered expression in endogenous SRA knockdown versus control 3T3-L1 adipocytes.

| GO BP ID | Pvalue | ExpCount | Count | Size | Term |
| --- | --- | --- | --- | --- | --- |
| GO:0007155 | 0 | 8 | 21 | 278 | cell adhesion |
| GO:0006817 | 0 | 1 | 6 | 28 | phosphate transport |
| GO:0030199 | 0 | 0 | 4 | 12 | collagen fibril organization |
| GO:0007169 | 0 | 3 | 10 | 92 | transmembrane receptor protein tyrosine kinase signaling pathway |
| GO:0048675 | 0 | 0 | 4 | 13 | axon extension |
| GO:0007275 | 0 | 28 | 45 | 978 | multicellular organismal development |
| GO:0009888 | 0.001 | 4 | 12 | 143 | tissue development |
| GO:0048518 | 0.002 | 13 | 25 | 461 | positive regulation of biological process |
| GO:0007399 | 0.002 | 9 | 19 | 313 | nervous system development |
| GO:0044236 | 0.003 | 0 | 3 | 10 | multicellular organismal metabolic process |
| GO:0007165 | 0.003 | 34 | 50 | 1174 | signal transduction |
| GO:0035295 | 0.003 | 3 | 9 | 101 | tube development |
| GO:0043062 | 0.004 | 2 | 6 | 53 | extracellular structure organization and biogenesis |
| GO:0051239 | 0.005 | 4 | 10 | 131 | regulation of multicellular organismal process |
| GO:0007186 | 0.005 | 6 | 13 | 199 | G-protein coupled receptor protein signaling pathway |
| GO:0007188 | 0.008 | 1 | 4 | 28 | G-protein signaling, coupled to cAMP nucleotide second messenger |
| GO:0006956 | 0.009 | 0 | 3 | 15 | complement activation |
| GO:0006633 | 0.009 | 1 | 5 | 45 | fatty acid biosynthetic process |
