# Supplementary material for: Multiple Roles for the Non-Coding RNA SRA in Regulation of Adipogenesis and Insulin Sensitivity
Source: PLoS One. 2010 Dec 2;5(12):e14199. doi: 10.1371/journal.pone.0014199 (PMC2996286; doi:10.1371/journal.pone.0014199)
Supplement: Table S4 — GO terms in molecular function (MF) overrepresented amongst genes with altered expression in endogenous SRA knockdown versus control 3T3-L1 adipocytes. (0.04 MB DOC) [file pone.0014199.s007.doc]

**Table S4**. GO terms in molecular function (MF) overrepresented amongst genes with altered expression in endogenous SRA knockdown versus control 3T3-L1 adipocytes.

| GO MF ID | Pvalue | ExpCount | Count | Size | Term |
| --- | --- | --- | --- | --- | --- |
| GO:0004872 | 0 | 17 | 34 | 588 | receptor activity |
| GO:0048503 | 0 | 1 | 8 | 51 | GPI anchor binding |
| GO:0019199 | 0 | 1 | 6 | 36 | transmembrane receptor protein kinase activity |
| GO:0060089 | 0.001 | 20 | 35 | 691 | molecular transducer activity |
| GO:0005543 | 0.001 | 3 | 10 | 101 | phospholipid binding |
| GO:0030020 | 0.001 | 1 | 4 | 18 | extracellular matrix structural constituent conferring tensile strength |
| GO:0016641 | 0.003 | 0 | 3 | 11 | oxidoreductase activity, acting on the CH-NH2 group of donors, oxygen as acceptor |
| GO:0004930 | 0.005 | 3 | 9 | 115 | G-protein coupled receptor activity |
| GO:0017048 | 0.007 | 0 | 3 | 14 | Rho GTPase binding |
| GO:0019899 | 0.007 | 2 | 7 | 79 | enzyme binding |
| GO:0005509 | 0.008 | 11 | 20 | 393 | calcium ion binding |
