# Supplementary material for: Multiple Roles for the Non-Coding RNA SRA in Regulation of Adipogenesis and Insulin Sensitivity
Source: PLoS One. 2010 Dec 2;5(12):e14199. doi: 10.1371/journal.pone.0014199 (PMC2996286; doi:10.1371/journal.pone.0014199)
Supplement: Table S5 — Down-regulated gene sets by SRA overexpression in ST2 cells analyzed by GSEA (0.22 MB DOC) [file pone.0014199.s008.doc]

**Table S5.** Down-regulated gene sets by SRA overexpression in ST2 cells analyzed by GSEA

| **NAME** | **SIZE** | **ES** | **NES** | **NOM**  **p-val** | **FDR q-val** | **FWER p-val** |
| --- | --- | --- | --- | --- | --- | --- |
| SERUM_FIBROBLAST_CELLCYCLE | 111 | 0.76 | 2.73 | 0 | 0 | 0 |
| LE_MYELIN_UP | 104 | 0.77 | 2.73 | 0 | 0 | 0 |
| IDX_TSA_UP_CLUSTER3 | 83 | 0.80 | 2.70 | 0 | 0 | 0 |
| BOQUEST_CD31PLUS_VS_CD31MINUS_DN | 232 | 0.64 | 2.50 | 0 | 0 | 0 |
| PRMT5_KD_UP | 169 | 0.66 | 2.48 | 0 | 0 | 0 |
| BRENTANI_CELL_CYCLE | 79 | 0.72 | 2.46 | 0 | 0 | 0 |
| LEE_TCELLS3_UP | 82 | 0.73 | 2.44 | 0 | 0 | 0 |
| IGLESIAS_E2FMINUS_UP | 145 | 0.66 | 2.44 | 0 | 0 | 0 |
| NEMETH_TNF_UP | 91 | 0.70 | 2.43 | 0 | 0 | 0 |
| GOLDRATH_CELLCYCLE | 32 | 0.84 | 2.43 | 0 | 0 | 0 |
| ZHAN_MM_CD138_PR_VS_REST | 34 | 0.82 | 2.39 | 0 | 0 | 0 |
| HOFFMANN_BIVSBII_BI_TABLE2 | 237 | 0.61 | 2.38 | 0 | 0 | 0 |
| GREENBAUM_E2A_UP | 32 | 0.81 | 2.37 | 0 | 0 | 0 |
| JECHLINGER_EMT_UP | 51 | 0.75 | 2.37 | 0 | 0 | 0 |
| IRITANI_ADPROX_DN | 53 | 0.74 | 2.37 | 0 | 0 | 0 |
| EMT_UP | 55 | 0.74 | 2.36 | 0 | 0 | 0 |
| P21_P53_ANY_DN | 40 | 0.79 | 2.36 | 0 | 0 | 0 |
| IRS1_KO_ADIP_DN | 104 | 0.67 | 2.34 | 0 | 0 | 0 |
| DER_IFNB_UP | 81 | 0.68 | 2.34 | 0 | 0 | 0 |
| CORDERO_KRAS_KD_VS_CONTROL_UP | 72 | 0.70 | 2.33 | 0 | 0 | 0 |
| DNMT1_KO_UP | 69 | 0.70 | 2.33 | 0 | 0 | 0 |
| CROONQUIST_IL6_STARVE_UP | 33 | 0.83 | 2.33 | 0 | 0 | 0 |
| CMV_HCMV_TIMECOURSE_24HRS_DN | 36 | 0.78 | 2.33 | 0 | 0 | 0 |
| CANCER_UNDIFFERENTIATED_META_UP | 64 | 0.71 | 2.33 | 0 | 0 | 0 |
| HCC_SURVIVAL_GOOD_VS_POOR_DN | 113 | 0.65 | 2.32 | 0 | 0 | 0 |
| IDX_TSA_DN_CLUSTER2 | 61 | 0.72 | 2.32 | 0 | 0 | 0 |
| BRCA_PROGNOSIS_NEG | 84 | 0.68 | 2.32 | 0 | 0 | 0 |
| ADIP_VS_PREADIP_DN | 36 | 0.79 | 2.32 | 0 | 0 | 0 |
| IRITANI_ADPROX_VASC | 137 | 0.64 | 2.31 | 0 | 0 | 0 |
| CMV_HCMV_TIMECOURSE_ALL_DN | 357 | 0.57 | 2.31 | 0 | 0 | 0 |
| LI_FETAL_VS_WT_KIDNEY_DN | 143 | 0.62 | 2.30 | 0 | 0 | 0 |
| POD1_KO_UP | 362 | 0.56 | 2.30 | 0 | 0 | 0 |
| DOX_RESIST_GASTRIC_UP | 32 | 0.80 | 2.29 | 0 | 0 | 0 |
| ADIP_DIFF_CLUSTER5 | 35 | 0.78 | 2.29 | 0 | 0 | 0 |
| GAY_YY1_DN | 271 | 0.58 | 2.27 | 0 | 0 | 0 |
| SANA_TNFA_ENDOTHELIAL_UP | 68 | 0.69 | 2.27 | 0 | 0 | 0 |
| IDX_TSA_DN_CLUSTER1 | 40 | 0.74 | 2.27 | 0 | 0 | 0 |
| P21_P53_MIDDLE_DN | 22 | 0.85 | 2.25 | 0 | 0 | 0 |
| YU_CMYC_UP | 41 | 0.76 | 2.25 | 0 | 0 | 0 |
| IFN_BETA_UP | 60 | 0.69 | 2.24 | 0 | 0 | 0 |
| TGFBETA_ALL_UP | 75 | 0.67 | 2.24 | 0 | 0 | 0 |
| SHEPARD_CRASH_AND_BURN_MUT_VS_WT_DN | 145 | 0.61 | 2.24 | 0 | 0 | 0 |
| SHEPARD_CELL_PROLIFERATION | 184 | 0.58 | 2.24 | 0 | 0 | 0 |
| IFNA_HCMV_6HRS_UP | 42 | 0.74 | 2.23 | 0 | 0 | 0 |
| SHEPARD_BMYB_MORPHOLINO_DN | 162 | 0.60 | 2.22 | 0 | 7.09E-05 | 0.002 |
| OLDAGE_DN | 45 | 0.71 | 2.22 | 0 | 6.93E-05 | 0.002 |
| CELL_PROLIFERATION | 184 | 0.58 | 2.22 | 0 | 6.79E-05 | 0.002 |
| ELONGINA_KO_UP | 155 | 0.59 | 2.21 | 0 | 1.31E-04 | 0.004 |
| CELL_CYCLE | 72 | 0.66 | 2.20 | 0 | 1.60E-04 | 0.005 |

(Continued on next page)

**Table S5, continued 1**

| GALINDO_ACT_UP | 78 | 0.65 | 2.20 | 0 | 1.88E-04 | 0.006 |
| --- | --- | --- | --- | --- | --- | --- |
| IRITANI_ADPROX_UP | 21 | 0.82 | 2.19 | 0 | 1.84E-04 | 0.006 |
| CMV_HCMV_6HRS_DN | 51 | 0.69 | 2.19 | 0 | 1.81E-04 | 0.006 |
| CELL_CYCLE_KEGG | 80 | 0.64 | 2.18 | 0 | 2.39E-04 | 0.008 |
| CMV_ALL_DN | 93 | 0.64 | 2.18 | 0 | 2.34E-04 | 0.008 |
| CROONQUIST_IL6_RAS_DN | 22 | 0.79 | 2.18 | 0 | 2.30E-04 | 0.008 |
| CMV_IE86_UP | 44 | 0.71 | 2.18 | 0 | 2.26E-04 | 0.008 |
| TGFBETA_EARLY_UP | 45 | 0.71 | 2.17 | 0 | 2.22E-04 | 0.008 |
| ADIP_VS_FIBRO_DN | 25 | 0.79 | 2.17 | 0 | 2.18E-04 | 0.008 |
| DSRNA_UP | 34 | 0.74 | 2.17 | 0 | 2.15E-04 | 0.008 |
| CMV_24HRS_DN | 67 | 0.66 | 2.17 | 0 | 2.37E-04 | 0.009 |
| IRS_KO_ADIP_DN | 38 | 0.74 | 2.17 | 0 | 2.33E-04 | 0.009 |
| IRITANI_ADPROX_LYMPH | 112 | 0.61 | 2.17 | 0 | 2.29E-04 | 0.009 |
| HDACI_COLON_BUT12HRS_DN | 61 | 0.67 | 2.16 | 0 | 2.76E-04 | 0.011 |
| PASSERINI_ADHESION | 36 | 0.73 | 2.15 | 0 | 2.97E-04 | 0.012 |
| P21_ANY_DN | 31 | 0.75 | 2.14 | 0 | 4.40E-04 | 0.018 |
| GENOTOXINS_ALL_4HRS_REG | 26 | 0.78 | 2.14 | 0 | 4.58E-04 | 0.019 |
| UVC_TTD_ALL_DN | 319 | 0.53 | 2.14 | 0 | 4.51E-04 | 0.019 |
| ADIP_DIFF_CLUSTER1 | 52 | 0.67 | 2.13 | 0 | 5.60E-04 | 0.023 |
| IDX_TSA_DN_CLUSTER3 | 75 | 0.64 | 2.13 | 0 | 5.52E-04 | 0.023 |
| ROS_MOUSE_AORTA_DN | 71 | 0.63 | 2.12 | 0 | 5.67E-04 | 0.024 |
| BREAST_DUCTAL_CARCINOMA_GENES | 19 | 0.83 | 2.12 | 0 | 5.59E-04 | 0.024 |
| GILDEA_BLADDER_UP | 25 | 0.77 | 2.12 | 0 | 5.51E-04 | 0.024 |
| UVC_HIGH_ALL_DN | 253 | 0.54 | 2.12 | 0 | 5.66E-04 | 0.025 |
| TARTE_PLASMA_BLASTIC | 277 | 0.53 | 2.11 | 0 | 6.86E-04 | 0.031 |
| AGEING_BRAIN_UP | 188 | 0.55 | 2.11 | 0 | 6.77E-04 | 0.031 |
| PEART_HISTONE_DN | 69 | 0.64 | 2.11 | 0 | 6.68E-04 | 0.031 |
| CANCER_NEOPLASTIC_META_UP | 60 | 0.65 | 2.11 | 0 | 7.21E-04 | 0.034 |
| IFN_ANY_UP | 76 | 0.63 | 2.11 | 0 | 7.12E-04 | 0.034 |
| ADIP_DIFF_CLUSTER4 | 33 | 0.74 | 2.11 | 0 | 7.03E-04 | 0.034 |
| H2O2_CSBRESCUED_UP | 55 | 0.66 | 2.10 | 0 | 8.92E-04 | 0.044 |
| TGFBETA_C2_UP | 17 | 0.83 | 2.10 | 0 | 9.20E-04 | 0.045 |
| BAF57_BT549_UP | 200 | 0.55 | 2.10 | 0 | 9.47E-04 | 0.046 |
| DIAB_NEPH_DN | 347 | 0.52 | 2.10 | 0 | 9.73E-04 | 0.048 |
| SCHRAETS_MLL_UP | 35 | 0.72 | 2.09 | 0 | 0.001 | 0.055 |
| GAMMA-UV_FIBRO_UP | 32 | 0.73 | 2.09 | 0 | 0.001 | 0.055 |
| VERNELL_PRB_CLSTR1 | 54 | 0.67 | 2.09 | 0 | 0.001 | 0.059 |
| SMITH_HTERT_DN | 59 | 0.64 | 2.08 | 0 | 0.001 | 0.064 |
| WALLACE_JAK2_DIFF | 20 | 0.79 | 2.08 | 0 | 0.001 | 0.065 |
| PROLIFERATION_GENES | 331 | 0.51 | 2.08 | 0 | 0.001 | 0.066 |
| G1_TO_S_CELL_CYCLE_REACTOME | 66 | 0.64 | 2.08 | 0 | 0.001 | 0.067 |
| HTERT_DN | 61 | 0.65 | 2.07 | 0 | 0.001 | 0.07 |
| UVC_TTD_4HR_DN | 264 | 0.52 | 2.07 | 0 | 0.001 | 0.077 |
| CMV_UV-CMV_COMMON_HCMV_6HRS_DN | 25 | 0.75 | 2.06 | 0 | 0.002 | 0.084 |
| ALCALAY_AML_NPMC_UP | 121 | 0.58 | 2.06 | 0 | 0.001 | 0.084 |
| G1PATHWAY | 24 | 0.75 | 2.05 | 0 | 0.002 | 0.097 |
| ALCALAY_AML_NPMC_DN | 159 | 0.55 | 2.05 | 0 | 0.002 | 0.098 |
| MANALO_HYPOXIA_DN | 78 | 0.60 | 2.05 | 0 | 0.002 | 0.101 |
| HDACI_COLON_BUT16HRS_DN | 91 | 0.59 | 2.05 | 0 | 0.002 | 0.106 |
| RIBOSOMAL_PROTEINS | 72 | 0.61 | 2.04 | 0 | 0.002 | 0.112 |
| BRENTANI_CYTOSKELETON | 18 | 0.81 | 2.04 | 0 | 0.002 | 0.112 |

(Continued on next page)

**Table S5, continued 2**

| NADLER_OBESITY_UP | 56 | 0.64 | 2.04 | 0 | 0.002 | 0.118 |
| --- | --- | --- | --- | --- | --- | --- |
| NF90_DN | 35 | 0.70 | 2.04 | 0 | 0.002 | 0.125 |
| AGEING_KIDNEY_SPECIFIC_UP | 157 | 0.55 | 2.04 | 0 | 0.002 | 0.126 |
| SASAKI_TCELL_LYMPHOMA_VS_CD4_UP | 146 | 0.55 | 2.04 | 0 | 0.002 | 0.126 |
| LEI_MYB_REGULATED_GENES | 269 | 0.51 | 2.04 | 0 | 0.002 | 0.129 |
| PARP_KO_UP | 29 | 0.72 | 2.03 | 0 | 0.002 | 0.134 |
| SASAKI_ATL_UP | 146 | 0.55 | 2.03 | 0 | 0.002 | 0.136 |
| ET743_SARCOMA_72HRS_DN | 184 | 0.53 | 2.03 | 0 | 0.002 | 0.137 |
| BECKER_TAMOXIFEN_RESISTANT_DN | 43 | 0.67 | 2.03 | 0 | 0.002 | 0.141 |
| HG_PROGERIA_DN | 23 | 0.75 | 2.03 | 0.002 | 0.002 | 0.149 |
| UVB_NHEK4_6HRS_UP | 24 | 0.74 | 2.03 | 0 | 0.002 | 0.153 |
| FLECHNER_KIDNEY_TRANSPLANT_WELL_UP | 472 | 0.49 | 2.02 | 0 | 0.002 | 0.153 |
| MAMMARY_DEV_UP | 52 | 0.63 | 2.02 | 0 | 0.002 | 0.16 |
| IDX_TSA_DN_CLUSTER5 | 42 | 0.67 | 2.02 | 0 | 0.003 | 0.165 |
| CMV-UV_HCMV_6HRS_DN | 93 | 0.58 | 2.02 | 0 | 0.003 | 0.166 |
| H2O2_CSBRESCUED_C1_UP | 41 | 0.66 | 2.02 | 0 | 0.003 | 0.167 |
| NAB_LUNG_DN | 47 | 0.65 | 2.02 | 0 | 0.003 | 0.174 |
| YAGI_AML_PROG_FAB | 173 | 0.54 | 2.02 | 0 | 0.003 | 0.175 |
| ZUCCHI_EPITHELIAL_UP | 38 | 0.68 | 2.02 | 0 | 0.003 | 0.178 |
| VANTVEER_BREAST_OUTCOME_GOOD_VS_POOR_DN | 60 | 0.63 | 2.01 | 0 | 0.003 | 0.187 |
