# Supplementary material for: Multiple Roles for the Non-Coding RNA SRA in Regulation of Adipogenesis and Insulin Sensitivity
Source: PLoS One. 2010 Dec 2;5(12):e14199. doi: 10.1371/journal.pone.0014199 (PMC2996286; doi:10.1371/journal.pone.0014199)
Supplement: Table S6 — Up-regulated gene sets by SRA overexpression in ST2 cells analyzed by GSEA. (0.04 MB DOC) [file pone.0014199.s009.doc]

**Table S6.** Up-regulated gene sets by SRA overexpression in ST2 cells analyzed by GSEA.

| NAME | SIZE | ES | NES | NOM  p-val | FDR q-val | FWER  p-val |
| --- | --- | --- | --- | --- | --- | --- |
| TNFALPHA_ADIP_DN | 57 | -0.61 | -2.08 | 0 | 0.03 | 0.10 |
| ADIP_DIFF_UP | 66 | -0.54 | -1.93 | 0 | 0.07 | 0.49 |
| TGZ_ADIP_UP | 15 | -0.71 | -1.84 | 0.00 | 0.12 | 0.79 |
| IDX_TSA_UP_CLUSTER6 | 150 | -0.40 | -1.62 | 0.00 | 0.20 | 1.00 |
| TNFALPHA_TGZ_ADIP_DN | 27 | -0.56 | -1.62 | 0.03 | 0.21 | 1.00 |
| LEE_MYC_TGFA_DN | 57 | -0.51 | -1.72 | 0.01 | 0.22 | 0.98 |
| LEE_DENA_DN | 64 | -0.46 | -1.62 | 0.01 | 0.23 | 1.00 |
| FETAL_LIVER_VS_ADULT_LIVER_GNF2 | 55 | -0.48 | -1.63 | 0.01 | 0.25 | 1.00 |
| CISPLATIN_PROBCELL_UP | 16 | -0.61 | -1.57 | 0.04 | 0.25 | 1.00 |
