# Supplementary material for: Multiple Roles for the Non-Coding RNA SRA in Regulation of Adipogenesis and Insulin Sensitivity
Source: PLoS One. 2010 Dec 2;5(12):e14199. doi: 10.1371/journal.pone.0014199 (PMC2996286; doi:10.1371/journal.pone.0014199)
Supplement: Table S7 — Down-regulated gene sets by SRA knockdown in 3T3-L1 cells analyzed by GSEA. (0.05 MB DOC) [file pone.0014199.s010.doc]

**Table S7.** Down-regulated gene sets by SRA knockdown in 3T3-L1 cells analyzed by GSEA.

| NAME | SIZE | ES | NES | NOM  p-val | FDR q-val | FWER  p-val |
| --- | --- | --- | --- | --- | --- | --- |
| POD1_KO_MOST_UP | 29 | 0.68 | 2.07 | 0 | 0.02 | 0.03 |
| MAMMARY_DEV_UP | 52 | 0.58 | 1.99 | 0 | 0.04 | 0.10 |
| IDX_TSA_DN_CLUSTER2 | 61 | 0.56 | 1.98 | 0 | 0.03 | 0.14 |
| EMT_UP | 55 | 0.55 | 1.94 | 0 | 0.05 | 0.23 |
| INSULIN_ADIP_INSENS_UP | 20 | 0.68 | 1.92 | 0 | 0.05 | 0.29 |
| N_GLYCAN_BIOSYNTHESIS | 21 | 0.66 | 1.85 | 0.002 | 0.09 | 0.54 |
| SPRYPATHWAY | 18 | 0.68 | 1.83 | 0.002 | 0.10 | 0.62 |
| JECHLINGER_EMT_UP | 51 | 0.53 | 1.83 | 0 | 0.09 | 0.63 |
| GH_EXOGENOUS_MIDDLE_UP | 89 | 0.47 | 1.77 | 0 | 0.17 | 0.87 |
| BOQUEST_CD31PLUS_VS_CD31MINUS_DN | 232 | 0.41 | 1.76 | 0 | 0.16 | 0.88 |
| PASSERINI_EM | 34 | 0.55 | 1.75 | 0.005 | 0.18 | 0.93 |
| XPB_TTD-CS_UP | 26 | 0.59 | 1.74 | 0.005 | 0.16 | 0.93 |
| ROSS_CBF_MYH | 44 | 0.53 | 1.73 | 0.003 | 0.18 | 0.96 |
| SCHRAETS_MLL_UP | 35 | 0.54 | 1.70 | 0.002 | 0.22 | 0.99 |
| ZHAN_MM_CD138_CD1_VS_REST | 37 | 0.53 | 1.70 | 0.006 | 0.21 | 0.99 |
