# Supplementary material for: Multiple Roles for the Non-Coding RNA SRA in Regulation of Adipogenesis and Insulin Sensitivity
Source: PLoS One. 2010 Dec 2;5(12):e14199. doi: 10.1371/journal.pone.0014199 (PMC2996286; doi:10.1371/journal.pone.0014199)
Supplement: Table S8 — Up-regulated gene sets by shSRA knockdown in 3T3-L1 cells analyzed by GSEA. (0.21 MB DOC) [file pone.0014199.s011.doc]

**Table S8.** Up-regulated gene sets by shSRA knockdown in 3T3-L1 cells analyzed by GSEA.

| NAME | SIZE | ES | NES | NOM p-val | FDR q-val | FWER p-val |
| --- | --- | --- | --- | --- | --- | --- |
| NADLER_OBESITY_DN | 35 | -0.67 | -2.29 | 0 | 0.002 | 0.001 |
| P21_P53_ANY_DN | 40 | -0.62 | -2.20 | 0 | 0.005 | 0.006 |
| ELECTRON_TRANSPORT_CHAIN | 91 | -0.49 | -2.06 | 0 | 0.027 | 0.052 |
| TGZ_ADIP_UP | 15 | -0.72 | -2.02 | 0 | 0.039 | 0.096 |
| ROS_MOUSE_AORTA_UP | 25 | -0.63 | -1.99 | 0 | 0.048 | 0.142 |
| LE_MYELIN_UP | 104 | -0.47 | -1.96 | 0 | 0.053 | 0.187 |
| ZHAN_MM_CD138_PR_VS_REST | 34 | -0.57 | -1.96 | 0 | 0.047 | 0.193 |
| BREAST_DUCTAL_CARCINOMA_GENES | 19 | -0.65 | -1.91 | 0 | 0.080 | 0.33 |
| MRNA_PROCESSING_REACTOME | 103 | -0.45 | -1.90 | 0 | 0.074 | 0.341 |
| P21_P53_MIDDLE_DN | 22 | -0.65 | -1.89 | 0 | 0.074 | 0.374 |
| MOOTHA_VOXPHOS | 76 | -0.47 | -1.89 | 0 | 0.073 | 0.397 |
| PEART_HISTONE_DN | 69 | -0.47 | -1.88 | 0 | 0.071 | 0.416 |
| ABBUD_LIF_UP | 46 | -0.51 | -1.87 | 0 | 0.071 | 0.443 |
| ADIP_VS_FIBRO_UP | 33 | -0.56 | -1.87 | 0 | 0.071 | 0.471 |
| YU_CMYC_UP | 41 | -0.53 | -1.87 | 0.003 | 0.067 | 0.473 |
| CITRATE_CYCLE_TCA_CYCLE | 17 | -0.65 | -1.86 | 0.005 | 0.064 | 0.483 |
| MRNA_PROCESSING | 40 | -0.51 | -1.86 | 0 | 0.062 | 0.493 |
| HUMAN_MITODB_6_2002 | 365 | -0.37 | -1.86 | 0 | 0.062 | 0.512 |
| HASLINGER_B_CLL_11Q23 | 18 | -0.64 | -1.84 | 0.005 | 0.071 | 0.586 |
| ADIP_DIFF_CLUSTER5 | 35 | -0.53 | -1.84 | 0.003 | 0.069 | 0.592 |
| HOUSTIS_ROS | 36 | -0.53 | -1.83 | 0 | 0.072 | 0.63 |
| 5FU_RESIST_GASTRIC_DN | 15 | -0.65 | -1.78 | 0.010 | 0.115 | 0.824 |
| IDX_TSA_DN_CLUSTER6 | 21 | -0.58 | -1.78 | 0.002 | 0.112 | 0.828 |
| MRNA_SPLICING | 44 | -0.50 | -1.77 | 0.003 | 0.116 | 0.855 |
| CPR_LOW_LIVER_DN | 22 | -0.56 | -1.76 | 0.008 | 0.122 | 0.875 |
| FETAL_LIVER_VS_ADULT_LIVER_GNF2 | 55 | -0.47 | -1.76 | 0.003 | 0.122 | 0.885 |
| SHIPP_DLBCL_CURED_UP | 24 | -0.56 | -1.75 | 0.010 | 0.120 | 0.888 |
| ADIP_DIFF_CLUSTER4 | 33 | -0.52 | -1.74 | 0.005 | 0.125 | 0.908 |
| MITOCHONDRIA | 372 | -0.34 | -1.74 | 0 | 0.123 | 0.909 |
| CELL_CYCLE_KEGG | 80 | -0.43 | -1.74 | 0.006 | 0.122 | 0.913 |
| UBIQUINONE_BIOSYNTHESIS | 15 | -0.65 | -1.74 | 0.014 | 0.118 | 0.913 |
| IDX_TSA_UP_CLUSTER3 | 83 | -0.42 | -1.74 | 0 | 0.116 | 0.915 |
| CELL_CYCLE | 72 | -0.43 | -1.73 | 0.003 | 0.118 | 0.922 |
| GOLDRATH_CELLCYCLE | 32 | -0.50 | -1.72 | 0 | 0.125 | 0.936 |
| RELAPATHWAY | 16 | -0.61 | -1.72 | 0.015 | 0.121 | 0.936 |
| AGEING_KIDNEY_DN | 101 | -0.40 | -1.71 | 0 | 0.124 | 0.945 |
| CMV_IE86_UP | 44 | -0.47 | -1.71 | 0.006 | 0.124 | 0.947 |
| NUMATA_G_CSF_DIFF | 18 | -0.59 | -1.70 | 0.025 | 0.130 | 0.96 |
| ADIP_VS_PREADIP_UP | 34 | -0.50 | -1.70 | 0.002 | 0.128 | 0.962 |
| P21_ANY_DN | 31 | -0.50 | -1.70 | 0.011 | 0.130 | 0.965 |
| BASSO_REGULATORY_HUBS | 122 | -0.39 | -1.70 | 0 | 0.128 | 0.966 |
| UNDERHILL_PROLIFERATION | 18 | -0.58 | -1.70 | 0.012 | 0.125 | 0.966 |
| CELL_CYCLE_REGULATOR | 23 | -0.54 | -1.70 | 0.007 | 0.122 | 0.966 |
| GLUTAMATE_METABOLISM | 24 | -0.54 | -1.68 | 0.012 | 0.131 | 0.977 |
| HIVNEFPATHWAY | 52 | -0.45 | -1.68 | 0 | 0.128 | 0.977 |

(Continued on next page)

**Table S8, Continued 1**

| PURINE_METABOLISM | 108 | -0.39 | -1.68 | 0 | 0.131 | 0.98 |
| --- | --- | --- | --- | --- | --- | --- |
| HESS_HOXAANMEIS1_UP | 70 | -0.42 | -1.67 | 0.006 | 0.139 | 0.988 |
| TNFALPHA_30MIN_UP | 39 | -0.47 | -1.66 | 0.005 | 0.142 | 0.989 |
| HESS_HOXAANMEIS1_DN | 70 | -0.42 | -1.65 | 0 | 0.151 | 0.993 |
| P53PATHWAY | 16 | -0.60 | -1.65 | 0.014 | 0.150 | 0.994 |
| DOX_RESIST_GASTRIC_UP | 32 | -0.49 | -1.65 | 0.007 | 0.150 | 0.994 |
| UVB_NHEK1_C6 | 115 | -0.38 | -1.64 | 0 | 0.151 | 0.995 |
| PRMT5_KD_UP | 169 | -0.36 | -1.64 | 0 | 0.150 | 0.995 |
| RIBOSOMAL_PROTEINS | 72 | -0.42 | -1.64 | 0 | 0.151 | 0.996 |
| EIF4PATHWAY | 24 | -0.51 | -1.63 | 0.020 | 0.152 | 0.997 |
| IDX_TSA_UP_CLUSTER5 | 90 | -0.39 | -1.63 | 0.006 | 0.157 | 0.998 |
| ETSPATHWAY | 17 | -0.56 | -1.63 | 0.019 | 0.157 | 0.998 |
| HDACI_COLON_BUT24HRS_UP | 59 | -0.42 | -1.62 | 0.009 | 0.161 | 1 |
| PARK_MSCS_DIFF | 32 | -0.48 | -1.61 | 0.025 | 0.173 | 1 |
| BADPATHWAY | 20 | -0.55 | -1.61 | 0.019 | 0.171 | 1 |
| ROSS_MLL_FUSION | 67 | -0.41 | -1.60 | 0.003 | 0.176 | 1 |
| PENG_GLUTAMINE_DN | 234 | -0.33 | -1.60 | 0 | 0.176 | 1 |
| IGF1PATHWAY | 20 | -0.53 | -1.60 | 0.032 | 0.174 | 1 |
| GLUTATHIONE_METABOLISM | 29 | -0.48 | -1.60 | 0.030 | 0.175 | 1 |
| RNA_TRANSCRIPTION_REACTOME | 36 | -0.46 | -1.59 | 0.013 | 0.177 | 1 |
| CHESLER_HIGHEST_FOLD_RANGE_GENES | 44 | -0.43 | -1.59 | 0.018 | 0.185 | 1 |
| TNFALPHA_ALL_UP | 70 | -0.39 | -1.58 | 0.009 | 0.192 | 1 |
| ADIP_DIFF_UP | 66 | -0.40 | -1.58 | 0.006 | 0.190 | 1 |
| IDX_TSA_UP_CLUSTER6 | 150 | -0.35 | -1.58 | 0 | 0.189 | 1 |
| GREENBAUM_E2A_UP | 32 | -0.46 | -1.56 | 0.015 | 0.208 | 1 |
| VEGF_MMMEC_3HRS_UP | 61 | -0.41 | -1.56 | 0.018 | 0.207 | 1 |
| FETAL_LIVER_ENRICHED_TRANSCRIPTION_FACTORS | 69 | -0.40 | -1.56 | 0.003 | 0.206 | 1 |
| HDACI_COLON_SUL48HRS_DN | 64 | -0.40 | -1.56 | 0.003 | 0.206 | 1 |
| NFKBPATHWAY | 23 | -0.51 | -1.55 | 0.026 | 0.212 | 1 |
| UVB_NHEK1_DN | 233 | -0.32 | -1.55 | 0 | 0.212 | 1 |
| TIDPATHWAY | 18 | -0.54 | -1.54 | 0.044 | 0.216 | 1 |
| GH_EXOGENOUS_EARLY_UP | 17 | -0.54 | -1.54 | 0.051 | 0.215 | 1 |
| HEARTFAILURE_ATRIA_UP | 23 | -0.50 | -1.54 | 0.037 | 0.217 | 1 |
| PGC | 323 | -0.31 | -1.54 | 0 | 0.214 | 1 |
| RADIATION_SENSITIVITY | 23 | -0.50 | -1.53 | 0.045 | 0.219 | 1 |
| H2O2_CSBDIFF_C1 | 28 | -0.47 | -1.53 | 0.035 | 0.217 | 1 |
| HISTIDINE_METABOLISM | 23 | -0.49 | -1.53 | 0.041 | 0.216 | 1 |
| PITX2PATHWAY | 16 | -0.54 | -1.53 | 0.041 | 0.216 | 1 |
| LEE_MYC_UP | 50 | -0.41 | -1.52 | 0.026 | 0.222 | 1 |
| BREASTCA_TWO_CLASSES | 126 | -0.35 | -1.52 | 0.003 | 0.221 | 1 |
| GENOTOXINS_24HRS_DISCR | 35 | -0.44 | -1.52 | 0.022 | 0.221 | 1 |
| AKTPATHWAY | 16 | -0.53 | -1.52 | 0.060 | 0.218 | 1 |
| LIN_WNT_UP | 49 | -0.41 | -1.52 | 0.025 | 0.218 | 1 |
| DNA_REPLICATION_REACTOME | 39 | -0.43 | -1.52 | 0.027 | 0.217 | 1 |
| G1_TO_S_CELL_CYCLE_REACTOME | 66 | -0.39 | -1.52 | 0.003 | 0.216 | 1 |
| HSP27PATHWAY | 15 | -0.55 | -1.52 | 0.051 | 0.217 | 1 |
| HBX_HEP_UP | 17 | -0.53 | -1.51 | 0.063 | 0.217 | 1 |
| HCC_SURVIVAL_GOOD_VS_POOR_DN | 113 | -0.35 | -1.51 | 0.003 | 0.219 | 1 |

(Continued on next page)

**Table S8, Continued 2**

| BRENTANI_CYTOSKELETON | 18 | -0.52 | -1.50 | 0.057 | 0.228 | 1 |
| --- | --- | --- | --- | --- | --- | --- |
| SIG_IL4RECEPTOR_IN_B_LYPHOCYTES | 26 | -0.47 | -1.50 | 0.050 | 0.228 | 1 |
| VALINE_LEUCINE_AND_ISOLEUCINE_DEGRADATION | 35 | -0.44 | -1.50 | 0.030 | 0.229 | 1 |
| IL2RBPATHWAY | 34 | -0.45 | -1.50 | 0.044 | 0.231 | 1 |
| CELL_CYCLE_CHECKPOINT | 24 | -0.49 | -1.49 | 0.061 | 0.231 | 1 |
| RACCYCDPATHWAY | 21 | -0.50 | -1.49 | 0.048 | 0.232 | 1 |
| HDACI_COLON_BUT16HRS_DN | 91 | -0.35 | -1.49 | 0.010 | 0.232 | 1 |
| AGED_MOUSE_CORTEX_UP | 31 | -0.45 | -1.49 | 0.049 | 0.230 | 1 |
| UVB_NHEK2_UP | 61 | -0.39 | -1.49 | 0.025 | 0.229 | 1 |
| HDACI_COLON_BUT48HRS_DN | 100 | -0.35 | -1.49 | 0.006 | 0.232 | 1 |
| MANALO_HYPOXIA_DN | 78 | -0.37 | -1.48 | 0.013 | 0.242 | 1 |
| TNFALPHA_ADIP_DN | 57 | -0.38 | -1.48 | 0.023 | 0.241 | 1 |
| BREASTCA_THREE_CLASSES | 37 | -0.42 | -1.47 | 0.040 | 0.244 | 1 |
| HSC_INTERMEDIATEPROGENITORS_SHARED | 110 | -0.34 | -1.47 | 0.010 | 0.244 | 1 |
| STRESS_ARSENIC_SPECIFIC_DN | 25 | -0.47 | -1.47 | 0.062 | 0.243 | 1 |
| UVC_HIGH_D9_DN | 20 | -0.49 | -1.47 | 0.068 | 0.243 | 1 |
| STRESSPATHWAY | 25 | -0.48 | -1.47 | 0.061 | 0.242 | 1 |
| IL7PATHWAY | 16 | -0.52 | -1.47 | 0.067 | 0.241 | 1 |
| 5FU_RESIST_GASTRIC_UP | 20 | -0.48 | -1.47 | 0.058 | 0.242 | 1 |
| BLEO_MOUSE_LYMPH_HIGH_24HRS_DN | 33 | -0.43 | -1.47 | 0.049 | 0.242 | 1 |
| TELPATHWAY | 15 | -0.53 | -1.46 | 0.082 | 0.243 | 1 |
| STURLA_SONIC_HEDGEHOG | 15 | -0.51 | -1.46 | 0.087 | 0.247 | 1 |
