# Supplementary material for: Multiple Roles for the Non-Coding RNA SRA in Regulation of Adipogenesis and Insulin Sensitivity
Source: PLoS One. 2010 Dec 2;5(12):e14199. doi: 10.1371/journal.pone.0014199 (PMC2996286; doi:10.1371/journal.pone.0014199)
Supplement: Table S9 — Primers used in real time PCR. (0.05 MB DOC) [file pone.0014199.s012.doc]

**Table S9.** Primers used in real time PCR.

| RNA RT-qPCR primers | Forward primer | Reverse primer |
| --- | --- | --- |
| mouse SRA | GGCTGGAGGGAAGTTGTCAATAC | CCACTGGTGATGTAAAAGTTCTTG |
| human SRA | TTGGAACAGGCATTGGAAGAC | ACAACTTTCCTCCAGCCCAC |
| PPARg | GGAAAGACAACGGACAAATCAC | TACGGATCGAAACTGGCAC |
| C/EBPa | TGAACAAGAACAGCAACGAG | TCACTGGTCACCTCCAGCAC |
| FABP4 | TGGAAGCTTGTCTCCAGTGA | AATCCCCATTTACGCTGATG |
| HSL | GGCAAAGAAGGATCGAAGAAC | TGTGTCATCGTGCGTAAATC |
| Sag | CAAGCATGAGGACACAAACC | GTGAGCTTCACCTTGATATGG |
| Fah | TTGGAACCACAATCTCCCCG | GTGGCAGAGATATGGCAAGG |
| CD36 | GAACAGCAGCAAAATCAAGG | AAGACACAGTGTGGTCCTC |
| PEPCK | TCGAAAGCAAGACAGTCATC | CAAAGTCCTCTTCCGACATC |
| Cyclophilin | TCCAAAGACAGCAGAAAACTTTCG | TCTTCTTGCTGGTCTTGCCATTCC |
| Sorbs1 | AGGAATCTCCGAAGCATGAAAG | TATAGCCCTTGGCAGCAAGTG |
| Socs3 | GCATCTTTGTCGGAAGACTG | AGTGGAGCATCATACTGATCC |
| Pkca | GAAAGCAACCATCCAACAAC | TCCTGTCAGCAAGCATCAC |
| Foxc2 | ACCTCCTGGTATCTGAACCAC | TGCGAGTTGAACATCTCCCG |
| Ube2c | TCTGGTGACAAAGGAATCTCC | ACCTCAGGTCTTCATATACGG |
| Ccna2 | AAAACCACTGACACCTCTTG | TCATTAACGTTCACTGGCTTG |
| Ccnb1-rs1 | TCCTCCGTAGAGCATCTAAAG | AAATGCACCATGTCGTAGTC |
| Bub1 | GGAATGGTTCAAGGAACACC | GAAGTGTTGGAGCCTGAAAC |
| Mcm5 | ACATCCAGGTCATGCTCAAG | GGCTGAAATGATGATGCCAG |
| Cdc20 | TCAACATCAAGGCGCTGTC | ACGTTCCAAATGCGAATGTG |
| Ncapd2 | GCCTTGCCATTGCTAACATC | CCTGAAGTCGCTCAAACAAC |
| Mki67 | CCTCAGATGGCTCAAAGAAC | AAAGAGATCCCCAGAAGTGG |
| Ccl2 | AGATGATCCCAATGAGTAGGC | CACAGACCTCTCTCTTGAGC |
| Ccl7 | CCCCAAGAGGAATCTCAAGAGC | TTCAGCGCAGACTTCCATGC |
| Kng1 | AGTAAAAAGTGCCCACAGAC | ATGGAGGGAAGGAAAACGC |
| Edg3 | TCTACTGCCTGGTCAAGTCC | GCAATGAACACACTCACCAC |
| Cxcl1 | GCTGGGATTCACCTCAAGAAC | AGCTTCAGGGTCAAGGCAAG |
| Tlr4 | CTGATCATGGCACTGTTCTTC | GCTGAGTTTCTGATCCATGC |
